# Supplementary figures and images for: Estimating quality-adjusted life expectancy (QALE) for local authorities in Great Britain and its association with indicators of the inclusive economy: a cross-sectional study
Source: BMJ Open. 2024 Mar 1;14(3):e076704. doi: 10.1136/bmjopen-2023-076704 (PMC10910677; doi:10.1136/bmjopen-2023-076704)

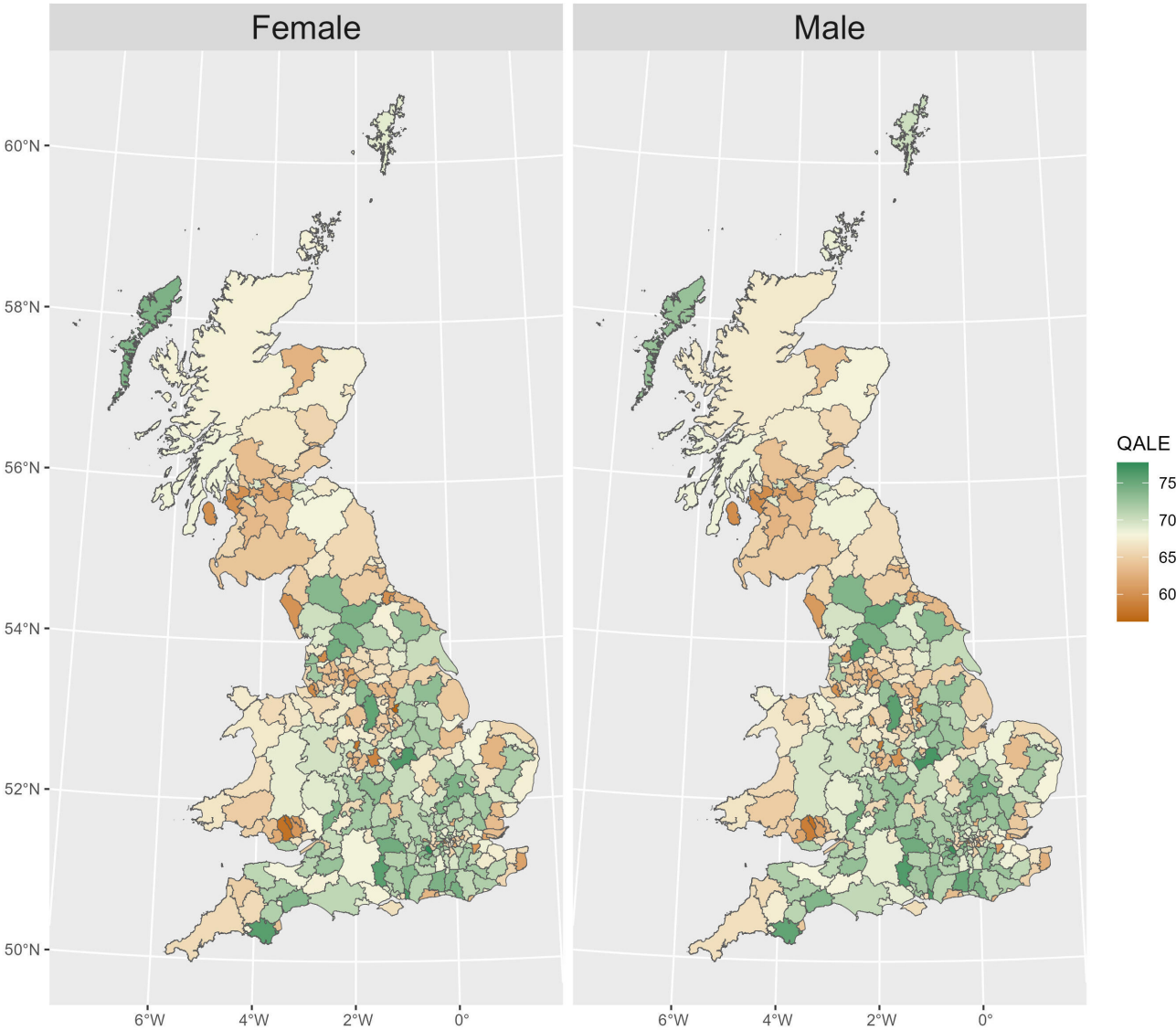

Supplement: Supplementary data [file bmjopen-2023-076704supp001.pdf]

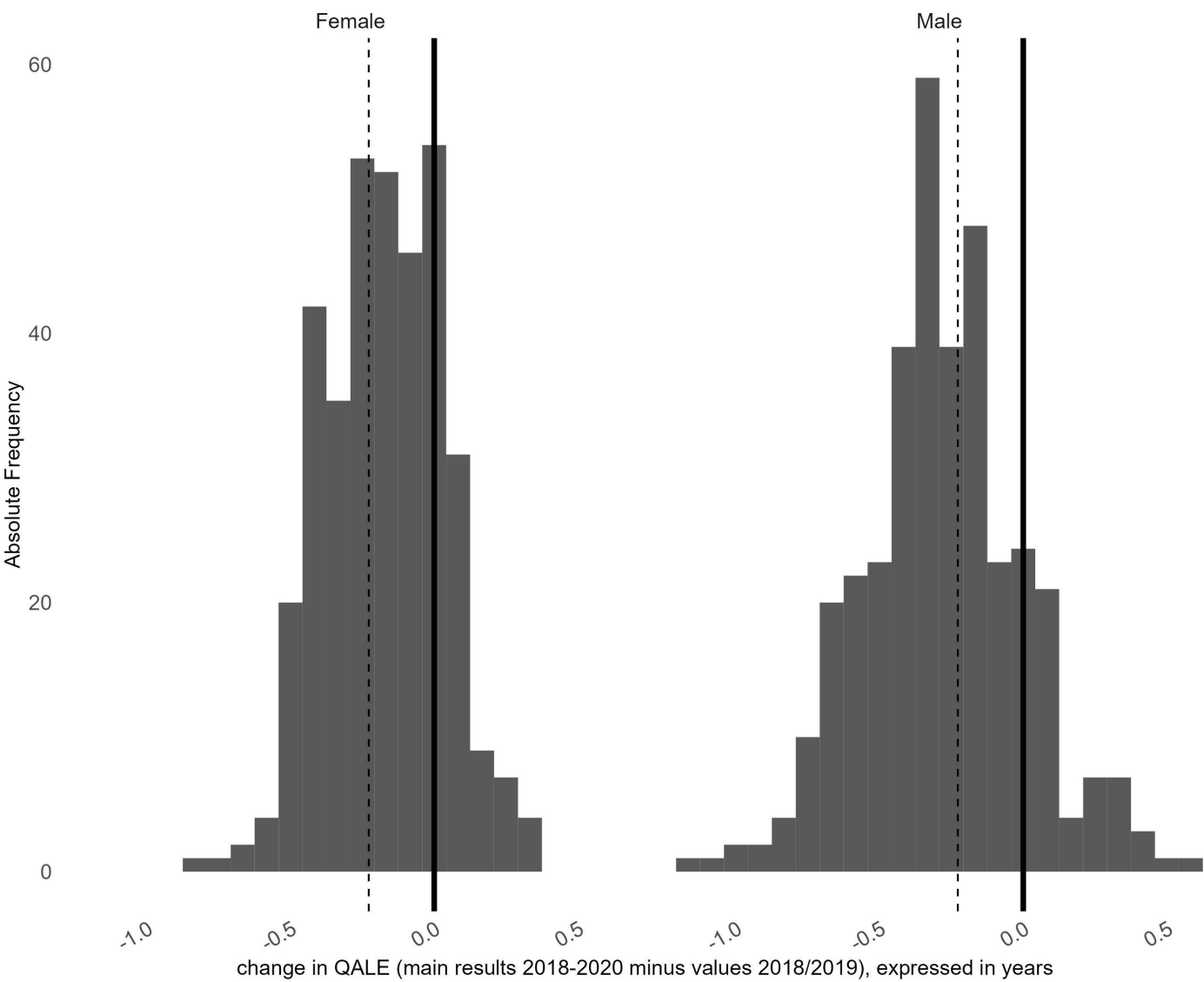

Supplement: Supplementary data [file bmjopen-2023-076704supp002.pdf]
