# Supplementary material for: Estimating quality-adjusted life expectancy (QALE) for local authorities in Great Britain and its association with indicators of the inclusive economy: a cross-sectional study
Source: BMJ Open. 2024 Mar 1;14(3):e076704. doi: 10.1136/bmjopen-2023-076704 (PMC10910677; doi:10.1136/bmjopen-2023-076704)
Supplement: Author's manuscript [file bmjopen-2023-076704.draft_revisions.pdf]

BMJ Open is committed to open peer review. As part of this commitment we make the peer review history of every article we publish publicly available.

When an article is published we post the peer reviewers' comments and the authors' responses online. We also post the versions of the paper that were used during peer review. These are the versions that the peer review comments apply to.

The versions of the paper that follow are the versions that were submitted during the peer review process. They are not the versions of record or the final published versions. They should not be cited or distributed as the published version of this manuscript.

BMJ Open is an open access journal and the full, final, typeset and author-corrected version of record of the manuscript is available on our site with no access controls, subscription charges or pay-per-view fees (http://bmjopen.bmj.com).

If you have any questions on BMJ Open's open peer review process please email info.bmjopen@bmj.com

BMJ Open

Estimating Quality-Adjusted Life Expectancy (QALE) for Local Authorities in Great Britain and its Association with Indicators of the Inclusive Economy: A Cross-Sectional Study.

Journal:	*BMJ Open*
Manuscript ID	bmjopen-2023-076704
Article Type:	Original research
Date Submitted by the Author:	14-Jun-2023

Complete List of Authors:	Höhn, Andreas; University of Glasgow, MRC/CSO Social and Public Health Sciences Unit<br>Lomax , Nik; University of Leeds, School of Geography<br>Rice, Hugh ; University of Leeds, School of Geography<br>Angus, Colin; University of Sheffield, School of Health and Related Research<br>Brennan, Alan; University of Sheffield, School of Health & Related Research<br>Brown, Denise ; University of Glasgow,<br>Cunningham, Anne; The University of Sheffield School of Health and Related Research<br>Elsenbroich, Corinna; University of Glasgow MRC/CSO Social and Public Health Sciences Unit<br>Hughes, Ceri; The University of Manchester, Manchester Institute of Education<br>Katikireddi, Srinivasa; University of Glasgow, MRC/CSO Social & Public Health Sciences Unit<br>McCartney, Gerry; University of Glasgow, Place and Wellbeing Directorate; Public Health Scotland Glasgow Office, Clinical and Protecting Health<br>Seaman, Rosie; University of Glasgow MRC/CSO Social and Public Health Sciences Unit<br>Tsuchia, Aki; University of Sheffield, Department of Economics<br>Meier, Petra; University of Glasgow MRC/CSO Social and Public Health Sciences Unit

Keywords:	Decision Making, HEALTH ECONOMICS, Health Equity, Quality of Life, PUBLIC HEALTH, STATISTICS & RESEARCH METHODS

SCHOLARONE™
Manuscripts

I, the Submitting Author has the right to grant and does grant on behalf of all authors of the Work (as defined in the below author licence), an exclusive licence and/or a non-exclusive licence for contributions from authors who are: i) UK Crown employees; ii) where BMJ has agreed a CC-BY licence shall apply, and/or iii) in accordance with the terms applicable for US Federal Government officers or employees acting as part of their official duties; on a worldwide, perpetual, irrevocable, royalty-free basis to BMJ Publishing Group Ltd ("BMJ") its licensees and where the relevant Journal is co-owned by BMJ to the co-owners of the Journal, to publish the Work in this journal and any other BMJ products and to exploit all rights, as set out in our licence.

The Submitting Author accepts and understands that any supply made under these terms is made by BMJ to the Submitting Author unless you are acting as an employee on behalf of your employer or a postgraduate student of an affiliated institution which is paying any applicable article publishing charge ("APC") for Open Access articles. Where the Submitting Author wishes to make the Work available on an Open Access basis (and intends to pay the relevant APC), the terms of reuse of such Open Access shall be governed by a Creative Commons licence – details of these licences and which Creative Commons licence will apply to this Work are set out in our licence referred to above.

Other than as permitted in any relevant BMJ Author's Self Archiving Policies, I confirm this Work has not been accepted for publication elsewhere, is not being considered for publication elsewhere and does not duplicate material already published. I confirm all authors consent to publication of this Work and authorise the granting of this licence.

For peer review only

Title Page

Title: Estimating Quality-Adjusted Life Expectancy (QALE) for Local Authorities in Great Britain and its Association with Indicators of the Inclusive Economy: A Cross-Sectional Study.

Author List: Andreas Höhn, Nik Lomax, Hugh Rice, Colin Angus, Alan Brennan, Denise Brown, Anne Cunningham, Corinna Elsenbroich, Ceri Hughes, Srinivasa Vittal Katikireddi, Gerry McCartney, Rosie Seaman, Aki Tsuchiya, and Petra Meier

Corresponding Author: Andreas Höhn, Research Associate in Public Health Equity, MRC/CSO Social and Public Health Sciences Unit, University of Glasgow, 99 Berkeley Street, Glasgow - G3 7HR, United Kingdom, email: andreas.hoehn(at)glasgow.ac.uk

Word count Abstract: 300/300 words

Word count paper: 4039 words

Number of References: 45

Number of figures: 4

Number of tables: 1

Funding/Support Statement: This work was supported by the UK Prevention Research Partnership (MR/S037578/2), which is funded by the British Heart Foundation, Cancer Research UK, Chief Scientist Office of the Scottish Government Health and Social Care Directorates, Engineering and Physical Sciences Research Council, Economic and Social Research Council, Health and Social Care Research and Development Division (Welsh Government), Medical Research Council, National Institute for Health Research, Natural Environment Research Council, Public Health Agency (Northern Ireland), The Health Foundation and Wellcome. In addition, this project received funding from the Medical Research Council (MC_UU_00022/5) as well as the Chief Scientist Office (SPHSU17 and SPHSU20). SVK acknowledges funding from the European Research Council (949582), and the Medical Research Council

For peer review only

(MC_UU_00022/2). The funders had no role in the design of the study or in the collection, analysis, and interpretation of data and results.

Competing interests Statement: no competing interests

Data Sharing Statement: We created a reproducibility pack for our analysis which includes code, data, and QALE estimates. The ***DOI*** will be provided after the peer review process.

Acknowledgment: We would like to thank Carl Schmertmann and Roland Rau for their strong commitment to open science, and Emma Comrie and Katherine Smith for providing comments on an earlier draft of this paper. We are very grateful for the opportunity to work with the UK Household Longitudinal Study (Understanding Society) and would like to thank the UK Data Service for granting special-license access to it.

Author Contributions: AH provided a first draft of the formal analysis and paper. AH, NL, HR and CH curated the underlying data. AH, NL, HR, and CH contributed to the formal data analysis and validation of results. AH, NL HR, CA, AB, DB, AC, CE, CH, SVK, GMC, RS, AT, and PM contributed to conceptualization, study design, interpretation of findings, and the discussion of implications. All authors contributed to the first draft of the paper, the revision of the paper, and have approved the final version. PM is the overall guarantor of this work.

For peer review only

For peer review only - http://bmjopen.bmj.com/site/about/guidelines.xhtml

Abstract

Objectives: Quantifying area-level inequalities in population health can help to inform policy responses. We describe an approach for estimating Quality-Adjusted Life Expectancy (QALE), a comprehensive health expectancy measure, for local authorities (LAs) in Great Britain (GB). To identify potential factors accounting for LA-level inequalities in QALE, we examined the association between inclusive economy indicators and QALE.

Setting: All 363 LAs in GB (lower tier/district level) within the period 2018-2020.

Data and Methods: We estimated lifetables for LAs using official statistics, and utility scores from the main-stage survey of Understanding Society. Using the Sullivan method, we estimated QALE at birth in years (y) with corresponding 80% Confidence Intervals (80% CIs). To examine the association between inclusive economy indicators and QALE, we used the inclusive economy indicator set of 13 variables, created by the System Science in Public Health and Health Economics Research (SIPHER) consortium.

Results: Overall, population-weighted QALE estimates across LAs in GB were the lowest in Scotland (females/males: 65.1y/64.9y) and Wales (65.1y/65.4y), while they were highest in England (67.6y/67.7y). The range across LAs for females, was from 56.3y (80% CI: 47.1 to 65.2) in Mansfield to 77.8y (69.1 to 86.4) in Runnymede. QALE for males ranged from 57.6y (44.6 to 70.7) in Merthyr Tydfil to 77.4y (71.6 to 83.3) in Runnymede. Indicators of the inclusive economy accounted for more than half of the variation in QALE at the LA level (adjusted R-squared females/males: 51%/58%). Although indicators of more inclusivity tended to be associated with higher levels of QALE at the LA level, this association was not consistent across all 13 indicators.

Conclusions: QALE can be estimated for LAs in GB, enabling further research into area-level health inequalities. The associations we identified between the inclusive economy indicators and QALE highlight potential policy priorities for improving population health and reducing health inequalities.

For peer review only - http://bmjopen.bmj.com/site/about/guidelines.xhtml

Strengths and limitations of this study

- We used a dedicated small-area approach to model mortality rates and health state utility scores - the two components required to estimate Quality-Adjusted Life Expectancy (QALE).
- The described small-area approach is suitable for estimating other health and state expectancy measures and is easily transferable to other national contexts for which similar data are available.
- As with most measures of health expectancy for small areas, QALE point estimates come with some degree of uncertainty, primarily due to their reliance on survey data.
- Differences in sampling strategies and non-response bias between local authorities in the Understanding Society survey might have contributed to differences in health state utility scores between areas.

For peer review only

Introduction

Period Life Expectancy (LE) is a summary measure of age-specific mortality rates, reflecting a particular point in time [1]. A limitation of LE is that it does not include any assessment of health. Health expectancy measures seek to address this limitation and capture both length of life and health status.

A variety of health expectancy measures are available for research and public health planning, including Healthy Life Expectancy (HLE), Disability-Free Life Expectancy (DFLE) – and more recently Quality Adjusted Life Expectancy (QALE). All three measures of health expectancy use lifetables to quantify the length of life [2,3]. However, these three health expectancy measures differ substantially in how they measure the health status of individuals. HLE and DFLE measure health in binary terms, by capturing individual-level health at every age as a value of either 1 *or* 0. HLE and DFLE reflect a single dimension of health (either “healthy” vs. ”unhealthy” or “without disability” vs. ”with disability”). QALE captures health in far greater detail using individual-level health state utility scores.

Individual-level utility scores measure health at every age as a value *between* 0 (dead) and 1 (perfect health) [3]. Individual-level utility scores are estimated from standardised questionnaires such as the EuroQol-5Dimensions (EQ-5D) or the ShortForm-12 (SF-12) - all of which capture a range of health dimensions [4,5]. Due to this greater detail, QALE captures both mental and physical health and is more sensitive to changes in health that would typically be masked by the binary definitions used for HLE or DFLE. This places QALE conceptually close to the gold standard measure, Quality-Adjusted Life Years (QALYs), which is the standard metric used in health economics to evaluate cost effectiveness [6].

A growing number of studies have used QALE to estimate the impact of health risk factors, shocks and interventions on population health. For example, QALE has been used as an outcome measure to understand the health burden of COVID-19 [7] and to shed light on the health impact of diseases [8,9] or adverse life course events [10]. QALE is also used in simulation studies to evaluate health technologies and policies

For peer review only - http://bmjopen.bmj.com/site/about/guidelines.xhtml

[11]. Despite its increasing popularity, QALE estimates are often not available for small areas. While QALE has been estimated for the general English population [12], and broken down by deprivation quintile [13], no QALE estimates are available for other GB nations or GB local authorities (LAs). In this paper, we present a small-area approach for estimating QALE for LAs (lower tier/district level) in England, Scotland, and Wales.

Despite continued economic growth, GB has experienced a stalling of improvements in mortality and health, alongside widening area-level inequalities [14]. In response, creating a more inclusive economy has increasingly gained traction as a suitable framework to tackle health inequalities [15]. In an exploratory analysis, we examined potential factors accounting for inequalities in QALE at the LA level using 13 inclusive economy indicators, created by the System Science in Public Health and Health Economics Research (SIPHER) consortium.

Data & Methods

Estimating Lifetables

QALE requires information on two components: mortality and health. To derive the mortality component, we estimated period lifetables based on mid-year population estimates and death counts. Mid-year population estimates for all three GB nations, as well as death counts for England and Wales, were accessed via the Office for National Statistics' (ONS) application programming interface (API) called NOMIS. Death counts for Scottish LAs in the respective period were obtained from the website of National Records for Scotland (NRS). For both the mortality and the health components of QALE, we consistently applied the ONS' definition of LA boundaries as of April 2021. We pooled data for the years 2018, 2019 and 2020. This period represented the most recent 3-year period for which harmonised mid-year population estimates were available.

For peer review only - http://bmjopen.bmj.com/site/about/guidelines.xhtml

Despite pooling data for three consecutive years, estimating robust age-specific mortality rates for an area with a small population size is challenging due to small and fluctuating numbers [16,17]. The potential imprecision in age-specific mortality rates increases as the size of the area decreases and fewer deaths are recorded [18]. One approach to address this challenge is to apply specific small-area estimation techniques, such as the tool for projecting age-specific rates using linear splines (TOPALS) models.

TOPALS models have previously been used to estimate mortality rates for small areas [19–22]. Given the general shape of a reference age pattern, TOPALS models estimate age-specific mortality rates by using penalised iteratively reweighted least squares (PIRLS). Reference age patterns should allow for smooth patterns [16] and align to the respective application [22]. We used data from the Human Mortality Database (HMD) for the general populations of England and Wales, and Scotland in the period 2010 to 2019 as a reference mortality schedule [23]. We corrected for potential over- or under-estimation of mortality after age 90 [24] - a phenomenon which can occur due to the functional form of TOPALS models [22].

We followed standard methodology to derive lifetables [25]. An example of sparse mortality data for LAs with small population sizes (e.g., males from the Shetland Islands) is presented in **Figure 1**. This shows raw death counts and raw death rates for 5-year age groups, and the modelled mortality rates for 1-year age groups.

[FIGURE 1 here]

Estimating Utility Scores

To derive the health component of QALE, we estimated age- and sex-specific utility scores using a special license version of the Understanding Society main stage survey. Expressed on a scale *between* 0 (dead) and 1 (perfect health), utility scores summarise an individual's health by combining information on physical and mental health

For peer review only - http://bmjopen.bmj.com/site/about/guidelines.xhtml

dimensions in one number [3,26]. Understanding Society is a large-scale, randomly sampled, panel survey which is representative of the UK population [27]. A special license was required to identify the LA of each interviewed household. We used wave *j*, which covered the period 2018-2020, the same period for which we estimated lifetables.

In Understanding Society, the SF-12 (Version 2) measure is used to capture the health of individuals. For each survey participant in Understanding Society, the SF-12 provides a summary score for the physical (PCS) and mental health (MCS) status. In wave *j*, SF-12 data were available for 16,481 females and 13,118 males.

Using cross-sectional survey weights, we calculated raw age-specific mean PCS scores and MCS scores, separately for males and females of each LA. However, as these raw age-and sex-specific mean PCS scores and MCS scores were generally noisy and not always available for all ages among some LAs with very low numbers of respondents, we applied a modelling approach.

Using two weighted linear regression models, we predicted age-specific mean SF-12 PCS scores and MCS scores for males and females in each LA from previously calculated raw age-and sex-specific mean PCS scores and MCS scores. Both models included covariates for; *sex* (female/male); *age* (categorical 10-year age groups allowing for non-linear age-trends); an *interaction effect between sex and age*; and a *fixed LA-effect* for each LA. We chose this functional form as it balanced statistical fit and over-fitting. All males and females aged 19 or younger were combined in the age group *0-19*. While the number of respondents varied across LAs, sample sizes were always sufficient for a fixed LA-effect to be estimated.

We converted all modelled age-and sex-specific mean SF-12 PCS scores and MCS scores into age-and sex-specific mean utility scores. For this purpose, we used the six-variable predictive model outlined by Lawrence and Fleishman [28] to map SF-12 PCS scores and MCS scores to utility scores. Conversions of SF-12 PCS scores and MCS scores into utility scores, via mapping, are common in health economics research with previous studies showing that mapping approaches generally perform well [29]. **Figure 2** shows the age- and sex-specific utility scores we estimated and highlights examples

For peer review only - http://bmjopen.bmj.com/site/about/guidelines.xhtml

for some of the largest LAs in GB and where authors were able to sense check results with SIPHER's local and national policy partners.

[FIGURE 2 here]

Quantifying Uncertainty of QALE Estimates

Our LA level QALE estimates are subject to some degree of uncertainty. To quantify the magnitude of this uncertainty, we provide confidence intervals (CIs) for all LA-level QALE point estimates. Across LAs, the number of survey participants was substantially lower when compared to the actual population size of the respective LA. We therefore estimated CIs for QALE point estimates based on the variance of the modelled age- and sex-specific utility scores [30]. This approach accounted for the number of individuals within an age group within each LA which were interviewed in the Understanding Society main stage survey. We provide 80% CIs, rather than 95% CIs. 80% CIs lead to smaller ranges and reflect 1.282 times the standard deviation as opposed 1.960 times the standard deviation. Using 80% CIs represents a compromise between uncertainty and usefulness with respect to our LA-level QALE estimates. 80% CIs have previously been used for health expectancy estimates for national, regional, and super-regional levels which are much larger geographies, with less uncertainty, than LAs [31].

Combining Lifetables and Utility Scores to Estimate QALE

We estimated QALE at birth for males and females for each LA using the Sullivan method [32]. The Sullivan method represents an adjustment of the lifetable parameter *nLx*, the number of person-years lived between age *x* and *x+1*, by the utility score of the age group *x*. Due to a very small number of respondents in the Understanding Society survey from the LAs *City of London* and *Isles of Scilly*, no QALE estimates were derived for these LAs. To ensure that geographical definitions are consistent across datasets

For peer review only - http://bmjopen.bmj.com/site/about/guidelines.xhtml

used in this study, we excluded these LAs from all analyses. This reduced the number of LAs for which QALE was estimated to 361 out of 363 LAs in GB.

Inclusive Economy Indicator Set

To identify potential factors accounting for QALE-inequalities, we examined the association between indicators of the inclusive economy and QALE. For this purpose, we merged QALE estimates with the inclusive economy indicator set, developed by the System Science in Public Health and Health Economics Research (SIPHER) consortium. SIPHER is a multi-disciplinary group of scientists and government partners at local, regional, and national levels [33].

To date, there is no single definition of the inclusive economy, making the concept challenging to operationalise. In response, SIPHER has created an inclusive economy indicator set for all 363 LAs in GB to provide a meaningful collection of data that can be used to explore the extent and nature of economic inclusion. Details of the iterative, co-production process for selecting final indicators have previously been described in a technical report [34]. From the inclusive economy indicator set, we used data for the year 2019, the midpoint of our three-year study period. A descriptive overview of all 13 inclusive economy indicators is presented in **Table 1**.

[TABLE 1 here]

We examined the association between indicators of the inclusive economy and QALE using multiple linear regression models. To ensure comparability across all 13 inclusive economy indicators, we used a z-transformation to standardise regression coefficients.

All analyses were performed in R (Version 4.3.0). NOMIS API queries were carried out using the NOMISR package [35]. TOPALS models followed the approach and software provided by Schmertmann & Rau [22,36].

For peer review only - http://bmjopen.bmj.com/site/about/guidelines.xhtml

Ethical Approval

The University of Essex Ethics Committee has approved all data collection for the Understanding Society main-stage survey and the specific special license linkage. We obtained a special license version of Understanding Society (PN 228941). No additional ethical approval was necessary for other data sources or analyses.

Patient and Public Involvement

We did not engage with patients and the public to comment on study design, define outcomes, conduct analyses, interpret results, or write the manuscript. However, co-production within the consortium, involving SIPHER's policy and practice partners, has contributed to different elements of our work on identifying and discussing indicators of the inclusive economy and measures of population health. Results presented in this paper surrounding QALE and indicators of the inclusive economy were presented and discussed at workshops with SIPHER's academic and policy partners. We will continue to disseminate the study results to our partners at local, regional, and national levels.

Results

National Differences in QALE

QALE differed between GB nations when comparing population-weighted averages across LAs. QALE was lowest in Scotland at levels of 65.1 years (y) among females and 64.9y among males. Levels were slightly higher in Wales with 65.1y among females and 65.4y among males. QALE was highest in England at levels of 67.6y among females and 67.7y among males.

For peer review only - http://bmjopen.bmj.com/site/about/guidelines.xhtml

For peer review only

Differences in QALE between Local Authorities

Visualising urban and rural areas with different population densities is challenging. Maps can be dominated by large rural areas, overshadowing small but densely populated areas and clustering into higher-level geographies. We therefore used hexagon cartograms to visualise the area-level distribution of QALE for females and males separately (**Figure 3**) [37]. This approach visually accounts for the population size of areas while retaining their contiguity and allowing for clustering into higher-level geographies.

For females in Scotland, we found QALE to be lowest in North Ayrshire at a level of 60.0y (80% CI: 53.2 to 66.6) and highest in Na h-Eileanan Siar at a level of 72.2y (66.7 to 78.8). Among males in Scotland, QALE was lowest in Renfrewshire at a level of 56.6y (53.6 to 65.7), while it was highest in Na h-Eileanan Siar at a level of 72.7y (66.7 to 78.8). This resulted in a difference of 12.2y (among females) and 16.1y (among males) in QALE point estimates between the Scottish LAs with the highest and the lowest level of QALE.

For females and males in Wales, we found QALE to be lowest in Merthyr Tydfil at levels of 57.0y (42.5 to 71.4) and 57.6y (44.6 to 70.7), while QALE was highest in Vale of Glamorgan at levels of 71.5y (65.6 to 77.5) and 71.4y (66.8 to 76.0), respectively. The corresponding difference in QALE point estimates between Welsh LAs with the highest and the lowest QALE was 14.5y among females and 13.8y among males.

Among females and males in England, QALE was lowest in Mansfield at levels of 56.4y (47.1 to 65.5) and 57.7y (49.5 to 65.9), while it was highest in Runnymede at levels of 77.8y (69.1 to 86.4) and 77.4y (71.6 to 83.3). Differences between the LAs with the highest and lowest QALE point estimates were the most striking in England at 21.4y difference among females and 19.7y difference among males.

Sex Differences in QALE within Local Authorities

When looking at differences between females and males within the same LA, there was always a clear female advantage in LE. However, there was not always a clear female

For peer review only - http://bmjopen.bmj.com/site/about/guidelines.xhtml

advantage in QALE. For example, females in Glasgow had a five year longer LE than males. The corresponding female QALE advantage was less than one year in Glasgow: 62.0y (57.8 to 66.3) among females and 61.2y (57.6 to 64.7) among males. For Sheffield, we found a female advantage in LE of approximately four years. However, there was a small disadvantage in QALE for females (63.4y (60.7 to 66.1)) when compared to males (63.8y (61.5 to 66.1)).

Associations between Inclusive Economy Indicators and QALE

We examined the association between the inclusive economy indicators and QALE to gain insights into potential factors contributing to QALE inequalities at the LA-level in GB (**Figure 4**). Overall, we found that inclusive economy indicators explained more than half of the variation in QALE between LAs in GB (adjusted R-squared females/males: 51%; 58%). We hypothesised that indicators reflecting more inclusivity would be associated with higher levels of QALE and that indicators reflecting lower levels of inclusivity would be associated with lower levels of QALE. As shown in **Figure 4**, our results were mixed as the direction of associations and levels of significance were not always consistent with our hypotheses.

For 7 out of the 13 inclusive economy indicators, we found the direction of the association between indicator and QALE to be in line with our hypotheses (more inclusive: higher QALE). For example, in relation to the observed mean level of public physical connectivity for the whole of GB (62.50%), an increase by 1 standard deviation (28.02%) was associated with an increase in QALE among males by 0.51y (95% CI: 0.16 to 0.87) and among females by 0.38y (95% CI: -0.01 to 0.76). Consistent with our hypotheses (less inclusive: lower QALE), we also found that an increase in digital exclusion by 1 standard deviation (20.22%), in relation to the observed GB mean level (33.86%), was associated with a decrease in QALE among males by 1.45y (95% CI: -1.97 to -0.92) and a decrease in QALE among females by 1.35y (95% CI: -1.91 to -0.79).

For the remaining 6 out of the 13 inclusive economy indicators, the direction of the association between indicator and QALE was not in line with what we hypothesised. For

For peer review only - http://bmjopen.bmj.com/site/about/guidelines.xhtml

example, we found that an increase in job security was associated with a decrease in QALE among males (-0.20y; 95% CI: -0.47 to 0.07) and females (-0.22y; 95% CI: -0.51 to 0.08).

One potential mechanism behind unexpected patterns could be residual confounding. For example, with respect to job security, non-permanent employment could be associated with well-paid occupations while high ratios of house prices to earnings (i.e., the metric used for the earnings inequality indicator) might be indicative of affluent suburban areas.

Discussion

Principal Findings

This paper details an approach for estimating QALE for LAs in GB, enabling further research into area-level health inequalities. Our approach harnesses the power of existing data and is easily transferable to other national contexts for which required data on mortality and health are available. Results of our exploratory analysis highlight the importance of economic inclusion as a potential policy priority for improving population health and reducing population health inequalities in GB.

Strengths and Limitations

This is the first study to estimate QALE for LAs in GB. Both the mortality and health components of QALE were derived via statistical modelling techniques, rather than being representative of raw input data only. We therefore consider our LA-level QALE estimates to be reliable, and our approach to be transferable to other national contexts for which respective small-area data is available.

All health expectancy measures, especially when estimated for areas with small population sizes, are subject to a degree of uncertainty. We have decided to not boost the sample size when estimating the health component of QALE - for example by using

For peer review only - http://bmjopen.bmj.com/site/about/guidelines.xhtml

a synthetic population dataset which would have made confidence intervals very small. Instead, we have relied on the original sample size and have reported 80% CI for all LA level QALE point estimates to provide a meaningful quantification of this uncertainty. Due to their reliance on survey data, the uncertainty surrounding health expectancy measures is generally much higher than the uncertainty surrounding corresponding LE estimates [38]. As a result, care is required when comparing health expectancy point estimates, between areas with small population sizes, such as rural LAs in GB and in longitudinal analyses using repeated cross-sectional snapshots.

To estimate lifetables, we used mid-year population estimates which are calculated using a cohort component model which rolls forward counts from the 2011 Census of population. Over time, unrecorded international migration, and migration between LAs could have had an impact on the quality of mid-year population estimates, affecting the estimation of age- and sex-specific mortality rates. As the civil registration system in GB does not allow us to follow individuals' LA of residence between censuses, this numerator-denominator issue is likely to remain an unresolved challenge for periods between censuses [39].

Understanding Society, the data source underlying the health component in our QALE estimates, is representative of the population of the UK [27]. However, sampling strategies as well as non-response are very likely to have varied across LAs, deprivation groups, and by health status. Weighting and oversampling are often unlikely to fully correct for all sampling limitations.

The data required to estimate QALE for small areas is often subject to safeguarded data access agreements, as was the case for Understanding Society. This presents challenges for reproducibility and open access.

Implications of Findings and Relevance to Other Studies

When aggregated and population-weighted, our QALE estimates for England are slightly lower than previously reported QALE estimates for the English population in

For peer review only - http://bmjopen.bmj.com/site/about/guidelines.xhtml

2017-2018 (females: 67.6y vs. 68.2y; males: 67.7y vs. 68.2y) [12]. Small differences in values were expected and could be due to differences in; the sampling biases in the survey data used, the measure used to capture health (EQ-5D vs. SF-12v2), or the years covered, with our QALE estimates including data for the first year of the COVID pandemic.

Our results indicate large inequalities in QALE across LAs in GB, further evidencing substantial area-level inequalities in population health. Our findings for QALE show several similarities when compared with patterns reported for HLE across LAs in GB [40,41]. For example, in line with results for HLE, the difference in QALE between areas with the highest and the lowest levels was much larger than the difference with respect to LE. This is further evidence that capturing mortality in isolation is likely to underestimate the differences in the burden of ill health experienced across GB.

Generally, we found areas with low levels of QALE to be among the lowest with respect to HLE. For example, we estimated QALE to be the lowest among males in Merthyr Tydfil at levels of 57.7y while HLE in the respective period was also low at a level of 58.9y [40]. Nevertheless, differences in how health is captured means that absolute levels and rankings will likely be inconsistent across different measures of health expectancy. At this point, research and policy would benefit from a comparative perspective, capturing the performance of LAs with respect to different health expectancy metrics such as LE, Lifespan Variation, DFLE, HLE, and QALE.

When looking within the same LA, females always had higher levels of LE, compared to males. In line with patterns for other health expectancy measures, such as HLE [42], the female advantage in QALE was very small, or negligible. This represents a health-survival paradox: females live longer but do not necessarily have better health in comparison to males. It is still not fully understood whether this health-survival paradox is the reflection of a true female health disadvantage (despite having a survival advantage) or the result of gender differences in self-reporting health [43]. It is possible that the health component of QALE could be affected by gender-specific survey response behaviour. Sex differences in QALE, could stem from gender differences in the SF-12 health instrument which we used to derive utility scores. The SF-12 health

For peer review only - http://bmjopen.bmj.com/site/about/guidelines.xhtml

instrument captures mental health explicitly. Averaged across LAs, we found the female disadvantage in the SF-12 mental health score (Females: 46.8 vs. Males: 49.2, $p<0.01$) to be larger than the female disadvantage in the SF-12 physical health score (Females: 48.7 vs. Males: 49.7, $p<0.01$).

Historically, improvements in population health were often attributed to economic growth [44]. However, this assumption is increasingly contested for high-income countries, including GB, as the association between economic growth and improving health does not seem to hold past certain levels of development [14,45]. In response, there have been growing calls to shift policy ambitions away from economic growth, towards inclusive economies, with a view to achieving more sustainable and equitable health improvements [15].

We found that inclusive economy indicators explained more than half of the variation in QALE between LAs in GB. This highlights the importance economic inclusion may have for improving population health and reducing area-level health inequalities. At the same time, associations found between inclusive economy indicators and QALE were not always consistent with our hypotheses, highlighting the concept’s complexity. One alternative approach would be to collapse information on all indicators into one single, averaging summary indicator, as it is often done with deprivation indices. However, one averaged summary indicator will likely fail to capture the diversity of inclusive economy indicators and the variation across different urban and rural areas. More work is needed to refine and test how the inclusive economy can be captured.

Conclusion

QALE is a comprehensive health expectancy measure and can be estimated for small areas in GB. QALE is conceptually close to the gold standard concept of QALYs, making it a comprehensive outcome measure for approaching area-level health inequalities, and evaluating past and future policies such as those seeking to establish more inclusive economies.

For peer review only - http://bmjopen.bmj.com/site/about/guidelines.xhtml

Tables

Table 1: Descriptive overview of Quality-Adjusted Life Expectancy (QALE) at birth, Life Expectancy (LE) at birth, and inclusive economy indicators for all Local Authorities (LAs) in England, Scotland, and Wales studied (n=361 LAs, meaning it does not consider the LAs City of London and Isles of Scilly). Note: Values in this table provided for the mean are representative of raw, unweighted averages across all studied LAs. In addition, please note the direction of indicators. For example, a higher employment rate is considered favourable, while a higher proportion of child poverty is less favourable. As some of the indicators reflect ratios, we did not change the direction of indicators. We utilised the direction provided in the underlying inclusive economy indicator set.

Indicator	Mean	SD	Min	Max	Explanation
LE Male	79.78	1.97	73.37	84.18	Life Expectancy
QALE Male	67.95	3.84	57.64	77.44	Quality-Adjusted Life Expectancy
LE Female	83.50	1.67	78.42	87.91	Life Expectancy
QALE Female	67.75	3.85	56.33	77.76	Quality-Adjusted Life Expectancy
Participation in paid employment	76.67	5.09	61.80	89.10	Percentage of working-age population (age 16-64) who are employed
Skills and qualifications	73.89	9.54	37.65	93.92	Percentage of adults aged 16-49 with a Level 2 or higher National Vocational Qualification (NVQ).
Involuntary exclusion from the labour market	4.97	2.15	1.05	11.74	Percentage of working-age population (age 16-64) who are economically inactive due to ill health or disability.
Digital exclusion	33.86	20.22	0.00	87.23	Percentage of individuals who are classified as e-withdrawn, passive and uncommitted internet users, or

For peer review only - http://bmjopen.bmj.com/site/about/guidelines.xhtml

Indicator	Mean	SD	Min	Max	Explanation
					settled offline communities -based on the Internet User Classification (IUC).
Wealth inequality	2.68	1.06	1.31	9.30	Ratio of max to min house sale prices within each local authority.
Physical connectivity	62.50	28.02	0.00	100.00	Proportion of LSOAs/DZs within the local authority area that are among the 50% most accessible LSOAs/DZs for each devolved nation (56%/44% split in England).
Earnings inequality	3.13	0.39	2.29	5.51	Ratio of weekly earnings (residents in full-time work) between 80th and 20th percentiles.
Housing affordability	9.92	3.62	3.95	34.46	Ratio of median house prices to median gross annual earnings (for residents) within each local authority.
Poverty	27.54	7.07	12.50	55.41	Percentage of children living in low-income households (after adjustment for housing costs).
Cost of living	10.83	2.59	5.56	21.63	Percentage of individuals reporting that they are worried about getting the food they need.
Decent Pay	21.77	6.06	7.50	40.40	Percentage of employee jobs that are not paid at or above the living wage.

For peer review only - http://bmjopen.bmj.com/site/about/guidelines.xhtml

Indicator	Mean	SD	Min	Max	Explanation
Inclusion in decision-making	35.18	6.19	21.60	64.47	Percentage of individuals participating in local elections out of all eligible voters.
Job security	95.31	1.69	86.13	98.33	Percentage of individuals in permanent employment out of all employed individuals aged 16-64.

For peer review only

References

1 Modig K, Rau R, Ahlbom A. Life expectancy: What does it measure? *BMJ open* 2020;**10**:e035932.

2 Gold MR, Stevenson D, Fryback DG. HALYS and QALYS and DALYS, oh my: Similarities and differences in summary measures of population health. *Annu Rev Public Health* 2002;**23**:115–34. doi:10.1146/annurev.publhealth.23.100901.140513

3 Prieto L, Sacristán JA. Problems and solutions in calculating quality-adjusted life years (QALYs). *Health and quality of life outcomes* 2003;**1**:1–8.

4 Sullivan PW, Slejko JF, Sculpher MJ, *et al.* Catalogue of EQ-5D scores for the united kingdom. *Med Decis Making* 2011;**31**:800–4. doi:10.1177/0272989X11401031

5 Brazier JE, Roberts J. The estimation of a preference-based measure of health from the SF-12. *Medical care* 2004;851–9.

6 Whitehead SJ, Ali S. Health outcomes in economic evaluation: The QALY and utilities. *British medical bulletin* 2010;**96**:5–21.

7 Wouterse B, Ram F, Baal P van. Quality-adjusted life-years lost due to COVID-19 mortality: Methods and application for the netherlands. *Value Health* 2022;**25**:731–5. doi:10.1016/j.jval.2021.12.008

8 Bronnum-Hansen H, Juel K, Davidsen M, *et al.* Impact of selected risk factors on quality-adjusted life expectancy in denmark. *Scand J Public Health* 2007;**35**:510–5. doi:10.1080/14034940701271908

9 Jia H, Zack MM, Thompson WW. The effects of diabetes, hypertension, asthma, heart disease, and stroke on quality-adjusted life expectancy. *Value Health* 2013;**16**:140–7. doi:10.1016/j.jval.2012.08.2208

10 Jia H, Lubetkin EI. Impact of adverse childhood experiences on quality-adjusted life expectancy in the u.s. population. *Child Abuse Negl* 2020;**102**:104418. doi:10.1016/j.chiabu.2020.104418

For peer review only

11 Health NI for, Excellence C. NICE health technology evaluations: The manual. Process and methods [PMG36]. 2022.https://www.nice.org.uk/guidance/pmg36/resources/nice-health-technology-evaluations-the-manual-pdf-72286779244741

12 McNamara S, Schneider PP, Love-Koh J, *et al.* Quality-adjusted life expectancy norms for the english population. *Value Health* Published Online First: 2022. doi:10.1016/j.jval.2022.07.005

13 Love-Koh J, Asaria M, Cookson R, *et al.* The social distribution of health: Estimating quality-adjusted life expectancy in england. *Value Health* 2015;**18**:655–62. doi:10.1016/j.jval.2015.03.1784

14 Walsh D, Dundas R, McCartney G, *et al.* Bearing the burden of austerity: How do changing mortality rates in the UK compare between men and women? *J Epidemiol Community Health* 2022;**76**:1027–33. doi:10.1136/jech-2022-219645

15 Shipton D, Sarica S, Craig N, *et al.* Knowing the goal: An inclusive economy that can address the public health challenges of our time. *J Epidemiol Community Health* 2021;**75**:1129–32. doi:10.1136/jech-2020-216070

16 Raalte AA van. What have we learned about mortality patterns over the past 25 years? *Population Studies* 2021;**75**:105–32.

17 Wilson T, Grossman I, Alexander M, *et al.* Methods for small area population forecasts: State-of-the-art and research needs. *Popul Res Policy Rev* 2022;**41**:865–98. doi:10.1007/s11113-021-09671-6

18 Eayres D, Williams ES. Evaluation of methodologies for small area life expectancy estimation. *J Epidemiol Community Health* 2004;**58**:243–9. doi:10.1136/jech.2003.009654

19 Beer J de. A new relational method for smoothing and projecting age-specific fertility rates: TOPALS. *Demographic Research* 2011;**24**:409–54. doi:10.4054/DemRes.2011.24.18

For peer review only

For peer review only - http://bmjopen.bmj.com/site/about/guidelines.xhtml

20 Beer J de. Smoothing and projecting age-specific probabilities of death by TOPALS. *Demographic Research* 2012;**27**:543–92. doi:10.4054/DemRes.2012.27.20

21 Gonzaga MR, Schmertmann CP. Estimating age- and sex-specific mortality rates for small areas with TOPALS regression: An application to brazil in 2010. *Revista Brasileira de Estudos de População* 2016;**33**:629–52. doi:10.20947/S0102-30982016c0009

22 Rau R, Schmertmann CP. District-level life expectancy in germany. *Dtsch Arztebl Int* 2020;**117**:493–9. doi:10.3238/arztebl.2020.0493

23 Barbieri M, Wilmoth JR, Shkolnikov VM, *et al.* Data resource profile: The human mortality database (HMD). *Int J Epidemiol* 2015;**44**:1549–56. doi:10.1093/ije/dyv105

24 Kannisto V, Lauritsen J, Thatcher AR, *et al.* Reductions in mortality at advanced ages: Several decades of evidence from 27 countries. *Population and development review* 1994;793–810.

25 Chiang CL. *Life table techniques and their applications*. Krieger Publishing Company 1984.

26 Kim YE, Jung YS, Ock M, *et al.* A review of the types and characteristics of healthy life expectancy and methodological issues. *J Prev Med Public Health* 2022;**55**:1–9. doi:10.3961/jpmph.21.580

27 Buck N, McFall S. Understanding society: Design overview. *Longitudinal and Life Course Studies* 2011;**3**:5–17.

28 Lawrence WF, Fleishman JA. Predicting EuroQoL EQ-5D preference scores from the SF-12 health survey in a nationally representative sample. *Med Decis Making* 2004;**24**:160–9. doi:10.1177/0272989X04264015

29 Chuang L-H, Kind P. Converting the SF-12 into the EQ-5D: An empirical comparison of methodologies. *Pharmacoeconomics* 2009;**27**:491–505.

For peer review only

30 Jagger C, Van Oyen H, Robine J-M. Health expectancy calculation by the sullivan method: A practical guide. 2014.

31 Permanyer I, Villavicencio F, Trias-Llimós S. Healthy lifespan inequality: Morbidity compression from a global perspective. *European Journal of Epidemiology* 2023;1–11.

32 Sullivan DF. A single index of mortality and morbidity. *HSMHA health reports* 1971;**86**:347.

33 Meier P, Purshouse R, Bain M, *et al.* The SIPHER consortium: Introducing the new UK hub for systems science in public health and health economic research. *Wellcome open research* 2019;**4**:174.

34 SIPHER. *The SIPHER inclusive economy indicator set: Technical paper*. SIPHER 2022. https://sipher.ac.uk/wp-content/uploads/2022/10/SIPHER-Inclusive-Economy-Indicator-set.pdf

35 Odell E. Nomisr: Access' nomis' UK labour market data. *Journal of Open Source Software* 2018;**3**:859.

36 Carl schmertmann: TOPALS fitting with age-grouped data. Https://github.com/schmert/TOPALS last accessed: 2023-05-10.

37 House of commons library: Geographical templates for non-contiguous cartograms of the UK. Https://github.com/houseofcommonslibrary/uk-hex-cartograms-noncontiguous last accessed: 2023-05-10.

38 Villavicencio F, Bergeron-Boucher M-P, Vaupel JW. Reply to permanyer et al.: The uncertainty surrounding healthy life expectancy indicators. *Proceedings of the National Academy of Sciences* 2021;**118**:e2115544118.

39 Leon DA, Shkolnikov VM. Widening life expectancy inequalities across small areas of england. *Lancet Public Health* 2021;**6**:e783–4. doi:10.1016/S2468-2667(21)00227-9

For peer review only

40 Office for national statistics: Health state life expectancies, UK: 2018 to 2020. Https://www.ons.gov.uk/peoplepopulationandcommunity/healthandsocialcare/healthandlifeexpectancies/bulletins/healthstatelifeexpectanciesuk/2018to2020 last accessed: 2023-05-10.

41 National records of scotland: Healthy life expectancy in scotland, 2018-2020. Https://www.nrscotland.gov.uk/statistics-and-data/statistics/statistics-by-theme/life-expectancy/healthy-life-expectancy-in-scotland/2018-2020 last accessed: 2023-05-10.

42 Jagger C. EU and UK targets for healthy life expectancy–are they achievable? *Vienna Yearbook of Population Research* 2021;**19**:15–22.

43 Oksuzyan A, Dańko MJ, Caputo J, *et al.* Is the story about sensitive women and stoical men true? Gender differences in health after adjustment for reporting behavior. *Social Science & Medicine* 2019;**228**:41–50.

44 Omran AR. The epidemiologic transition: A theory of the epidemiology of population change. 1971. *Milbank Q* 2005;**83**:731–57. doi:10.1111/j.1468-0009.2005.00398.x

45 Walsh D, Wyper GMA, McCartney G. Trends in healthy life expectancy in the age of austerity. *J Epidemiol Community Health* 2022;**76**:743–5. doi:10.1136/jech-2022-219011

For peer review only

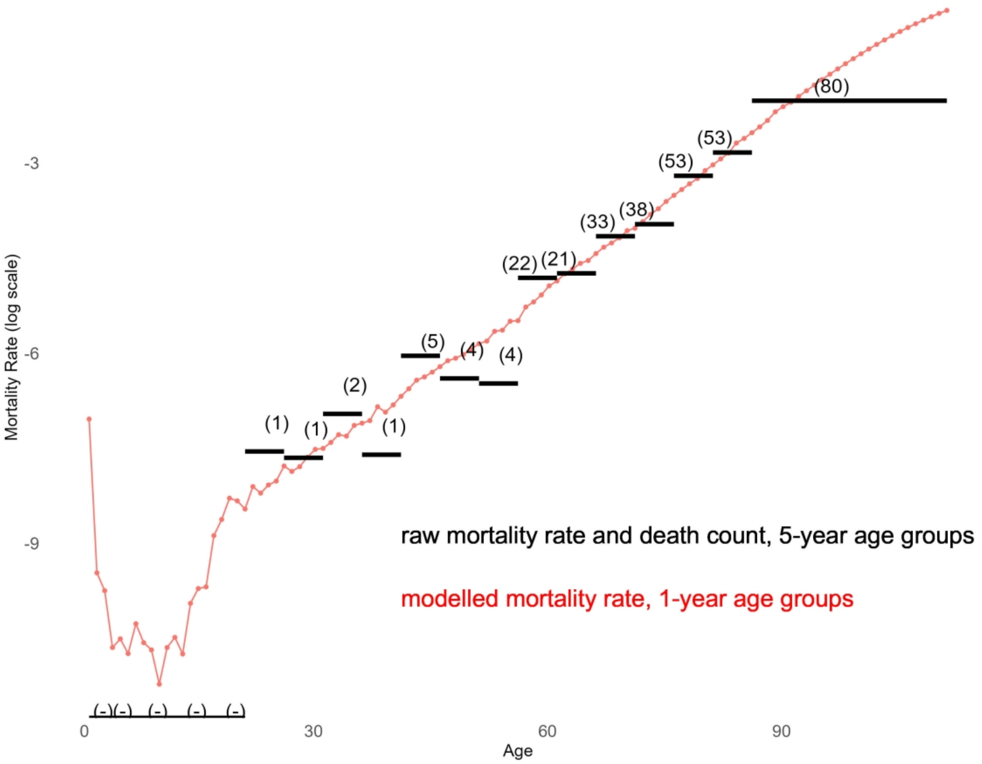

Figure 1: Number of observed deaths, the resulting raw death rate (log scale), and estimated mortality rates for males (log scale) of the Scottish local authority Shetland Islands, in 2018-2020.

1041x833mm (72 x 72 DPI)

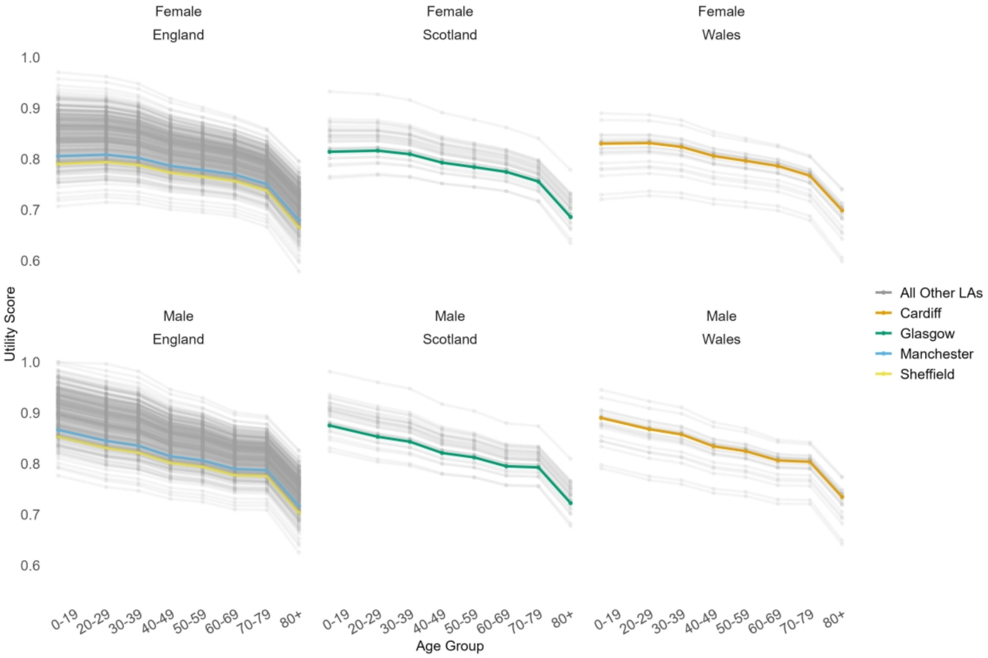

Figure 2: Utility scores obtained from SF12v2 PCS and MCS mean values for females and males in all local authorities in England, Scotland, and Wales with selected local authorities highlighted.

1249x833mm (72 x 72 DPI)

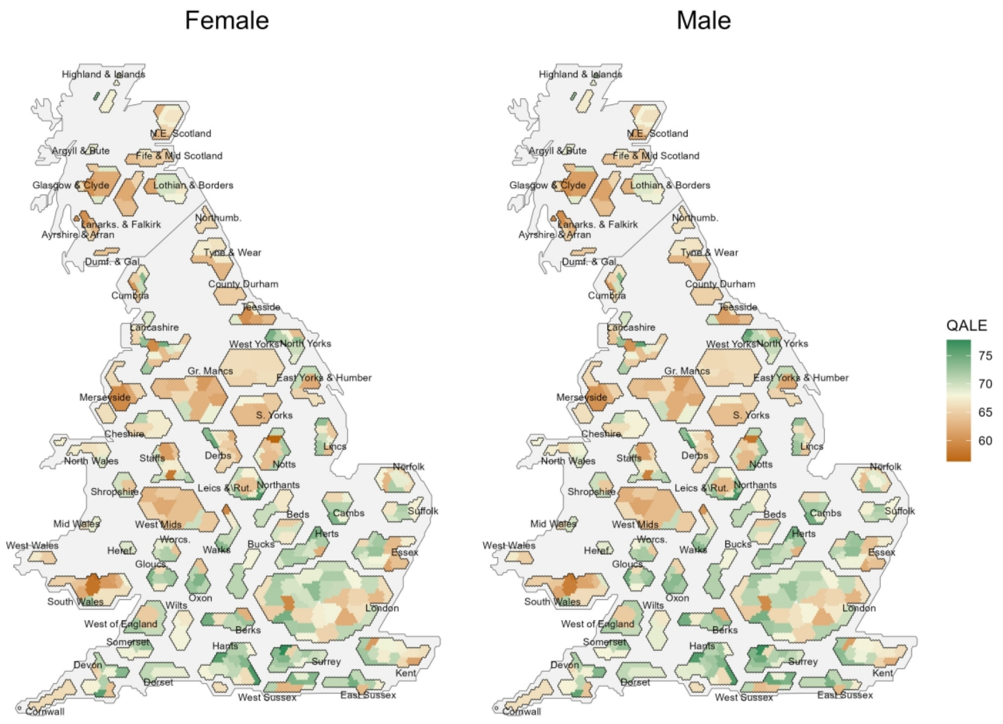

Figure 3: Quality-Adjusted Life Expectancy (QALE) at birth in years in 2018-2020 for females and males in local authorities in England, Scotland, and Wales. Note: the mid-point of the colour scale is referring to the unweighted mean in QALE across all GB LAs.

1041x833mm (72 x 72 DPI)

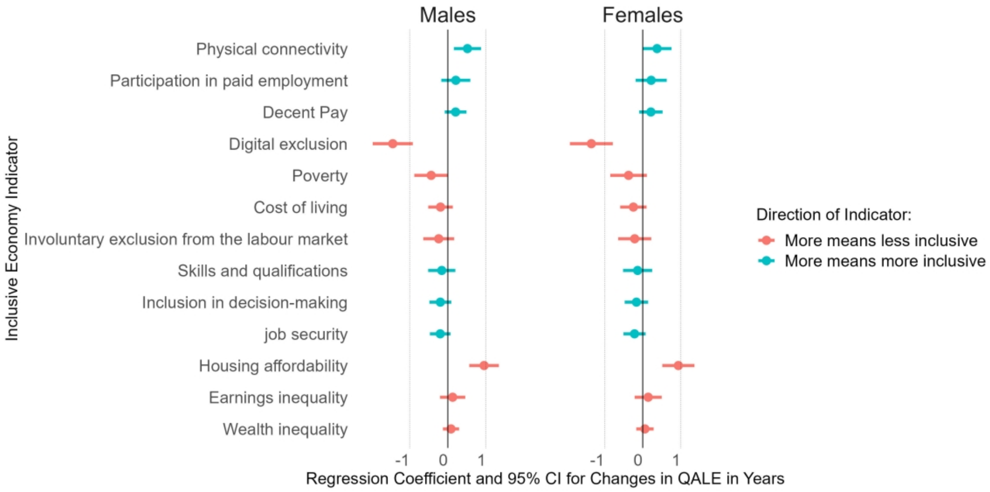

Figure 4: Multiple linear regression model for the association between inclusive economy indicators and Quality-Adjusted Life Expectancy (QALE) at birth in years and respective 95% confidence intervals (95% CIs). Note: regression coefficients were z-transformed. This means that regression coefficients show the expected change in QALE in years for a one standard deviation increase among the indicators, with respect to the GB mean level.

1249x624mm (72 x 72 DPI)

STROBE Statement—checklist of items that should be included in reports of observational studies

	Item No	Recommendation	Page No
**Title and abstract**	1	(*a*) Indicate the study's design with a commonly used term in the title or the abstract	1-2
		(*b*) Provide in the abstract an informative and balanced summary of what was done and what was found	3
**Introduction**			
Background/rationale	2	Explain the scientific background and rationale for the investigation being reported	5-6
Objectives	3	State specific objectives, including any prespecified hypotheses	5-6
**Methods**			
Study design	4	Present key elements of study design early in the paper	6
Setting	5	Describe the setting, locations, and relevant dates, including periods of recruitment, exposure, follow-up, and data collection	6-10
Participants	6	(*a*) *Cohort study*—Give the eligibility criteria, and the sources and methods of selection of participants. Describe methods of follow-up<br>*Case-control study*—Give the eligibility criteria, and the sources and methods of case ascertainment and control selection. Give the rationale for the choice of cases and controls<br>*Cross-sectional study*—Give the eligibility criteria, and the sources and methods of selection of participants	6-10
		(*b*) *Cohort study*—For matched studies, give matching criteria and number of exposed and unexposed<br>*Case-control study*—For matched studies, give matching criteria and the number of controls per case	NA
Variables	7	Clearly define all outcomes, exposures, predictors, potential confounders, and effect modifiers. Give diagnostic criteria, if applicable	6-10
Data sources/ measurement	8*	For each variable of interest, give sources of data and details of methods of assessment (measurement). Describe comparability of assessment methods if there is more than one group	*6-11*
Bias	9	Describe any efforts to address potential sources of bias	6-10
Study size	10	Explain how the study size was arrived at	6-10
Quantitative variables	11	Explain how quantitative variables were handled in the analyses. If applicable, describe which groupings were chosen and why	6-10
Statistical methods	12	(*a*) Describe all statistical methods, including those used to control for confounding	6-10
		(*b*) Describe any methods used to examine subgroups and interactions	6-10
		(*c*) Explain how missing data were addressed	6-10
		(*d*) *Cohort study*—If applicable, explain how loss to follow-up was addressed<br>*Case-control study*—If applicable, explain how matching of cases and controls was addressed<br>*Cross-sectional study*—If applicable, describe analytical methods taking account of sampling strategy	6-10
		(*e*) Describe any sensitivity analyses	NA

Continued on next page

For peer review only - http://bmjopen.bmj.com/site/about/guidelines.xhtml

**Results**			
Participants	13*	(a) Report numbers of individuals at each stage of study—eg numbers potentially eligible, examined for eligibility, confirmed eligible, included in the study, completing follow-up, and analysed	6-10
		(b) Give reasons for non-participation at each stage	NA
		(c) Consider use of a flow diagram	NA
Descriptive data	14*	(a) Give characteristics of study participants (eg demographic, clinical, social) and information on exposures and potential confounders	NA
		(b) Indicate number of participants with missing data for each variable of interest	NA
		(c) *Cohort study*—Summarise follow-up time (eg, average and total amount)	NA
Outcome data	15*	*Cohort study*—Report numbers of outcome events or summary measures over time	NA
		Case-control study—Report numbers in each exposure category, or summary measures of exposure	NA
		Cross-sectional study—Report numbers of outcome events or summary measures	11-14
Main results	16	(*a*) Give unadjusted estimates and, if applicable, confounder-adjusted estimates and their precision (eg, 95% confidence interval). Make clear which confounders were adjusted for and why they were included	11-14
		(*b*) Report category boundaries when continuous variables were categorized	11-13
		(*c*) If relevant, consider translating estimates of relative risk into absolute risk for a meaningful time period	NA
Other analyses	17	Report other analyses done—eg analyses of subgroups and interactions, and sensitivity analyses	NA
**Discussion**			
Key results	18	Summarise key results with reference to study objectives	14
Limitations	19	Discuss limitations of the study, taking into account sources of potential bias or imprecision. Discuss both direction and magnitude of any potential bias	14-15
Interpretation	20	Give a cautious overall interpretation of results considering objectives, limitations, multiplicity of analyses, results from similar studies, and other relevant evidence	15-17
Generalisability	21	Discuss the generalisability (external validity) of the study results	15-17
**Other information**			
Funding	22	Give the source of funding and the role of the funders for the present study and, if applicable, for the original study on which the present article is based	1-2

*Give information separately for cases and controls in case-control studies and, if applicable, for exposed and unexposed groups in cohort and cross-sectional studies.

Note: An Explanation and Elaboration article discusses each checklist item and gives methodological background and published examples of transparent reporting. The STROBE checklist is best used in conjunction with this article (freely available on the Web sites of PLoS Medicine at http://www.plosmedicine.org/, Annals of Internal Medicine at http://www.annals.org/, and Epidemiology at http://www.epidem.com/). Information on the STROBE Initiative is available at www.strobe-statement.org.

For peer review only

BMJ Open

Estimating Quality-Adjusted Life Expectancy (QALE) for Local Authorities in Great Britain and its Association with Indicators of the Inclusive Economy: A Cross-Sectional Study.

Journal:	*BMJ Open*
Manuscript ID	bmjopen-2023-076704.R1
Article Type:	Original research
Date Submitted by the Author:	07-Feb-2024

Complete List of Authors:	Höhn, Andreas; University of Glasgow, MRC/CSO Social and Public Health Sciences Unit<br>Lomax , Nik; University of Leeds, School of Geography<br>Rice, Hugh ; University of Leeds, School of Geography<br>Angus, Colin; University of Sheffield, School of Medicine and Population Health<br>Brennan, Alan; University of Sheffield, School of Medicine and Population Health<br>Brown, Denise ; University of Glasgow MRC/CSO Social and Public Health Sciences Unit<br>Cunningham, Anne; University of Sheffield, School of Medicine and Population Health<br>Elsenbroich, Corinna; University of Glasgow MRC/CSO Social and Public Health Sciences Unit<br>Hughes, Ceri; The University of Manchester, Manchester Institute of Education<br>Katikireddi, Srinivasa; University of Glasgow MRC/CSO Social and Public Health Sciences Unit<br>McCartney, Gerry; University of Glasgow School of Social and Political Sciences<br>Seaman, Rosie; University of Glasgow MRC/CSO Social and Public Health Sciences Unit<br>Tsuchia, Aki; University of Sheffield, School of Medicine and Population Health<br>Meier, Petra; University of Glasgow MRC/CSO Social and Public Health Sciences Unit

**Primary Subject Heading**:	Public health
Secondary Subject Heading:	Health economics, Research methods, Health policy
Keywords:	Decision Making, HEALTH ECONOMICS, Health Equity, Quality of Life, PUBLIC HEALTH, STATISTICS & RESEARCH METHODS

I, the Submitting Author has the right to grant and does grant on behalf of all authors of the Work (as defined in the below author licence), an exclusive licence and/or a non-exclusive licence for contributions from authors who are: i) UK Crown employees; ii) where BMJ has agreed a CC-BY licence shall apply, and/or iii) in accordance with the terms applicable for US Federal Government officers or employees acting as part of their official duties; on a worldwide, perpetual, irrevocable, royalty-free basis to BMJ Publishing Group Ltd ("BMJ") its licensees and where the relevant Journal is co-owned by BMJ to the co-owners of the Journal, to publish the Work in this journal and any other BMJ products and to exploit all rights, as set out in our licence.

The Submitting Author accepts and understands that any supply made under these terms is made by BMJ to the Submitting Author unless you are acting as an employee on behalf of your employer or a postgraduate student of an affiliated institution which is paying any applicable article publishing charge ("APC") for Open Access articles. Where the Submitting Author wishes to make the Work available on an Open Access basis (and intends to pay the relevant APC), the terms of reuse of such Open Access shall be governed by a Creative Commons licence – details of these licences and which Creative Commons licence will apply to this Work are set out in our licence referred to above.

Other than as permitted in any relevant BMJ Author's Self Archiving Policies, I confirm this Work has not been accepted for publication elsewhere, is not being considered for publication elsewhere and does not duplicate material already published. I confirm all authors consent to publication of this Work and authorise the granting of this licence.

For peer review only

Title Page

Title: Estimating Quality-Adjusted Life Expectancy (QALE) for Local Authorities in Great Britain and its Association with Indicators of the Inclusive Economy: A Cross-Sectional Study.

Author list: Andreas Höhn, Nik Lomax, Hugh Rice, Colin Angus, Alan Brennan, Denise Brown, Anne Cunningham, Corinna Elsenbroich, Ceri Hughes, Srinivasa Vittal Katikireddi, Gerry McCartney, Rosie Seaman, Aki Tsuchiya, and Petra Meier

Corresponding author: Andreas Höhn, Research Associate in Public Health Equity, MRC/CSO Social and Public Health Sciences Unit, University of Glasgow, 99 Berkeley Street, Glasgow - G3 7HR, United Kingdom

Corresponding author email address: andreas.hoehn@glasgow.ac.uk

Word count abstract: 299/300 words

Word count paper: 4731 words

Number of references: 53

Number of figures: 4

Number of tables: 1

For peer review only

Acknowledgment: This research was conducted as part of the Systems Science in Public Health and Health Economics Research - SIPHER Consortium and we thank the whole team for valuable input and discussions that have informed this work. We would like to thank Emma Comrie and Katherine Smith for providing comments on an earlier draft of this paper, as well as Carl Schmertmann and Roland Rau for their strong commitment to open science. We are very grateful for the opportunity to work with the UK Household Longitudinal Study (Understanding Society). Understanding Society is an initiative funded by the Economic and Social Research Council and various Government Departments, with scientific leadership by the Institute for Social and Economic Research, University of Essex, and survey delivery by the National Centre for Social Research (NatCen) and Verian (formerly Kantar Public). The research data are distributed by the UK Data Service.

For peer review only

Abstract

Objectives: Quantifying area-level inequalities in population health can help to inform policy responses. We describe an approach for estimating quality-adjusted life expectancy (QALE), a comprehensive health expectancy measure, for local authorities (LAs) in Great Britain (GB). To identify potential factors accounting for LA-level QALE inequalities, we examined the association between inclusive economy indicators and QALE.

Setting: 361/363 LAs in GB (lower tier/district level) within the period 2018-2020.

Data and Methods: We estimated life tables for LAs using official statistics, and utility scores from an area-level linkage of the Understanding Society survey. Using the Sullivan method, we estimated QALE at birth in years (y) with corresponding 80% Confidence Intervals (80% CIs). To examine the association between inclusive economy indicators and QALE, we used an open access data set operationalising the inclusive economy, created by the System Science in Public Health and Health Economics Research (SIPHER) consortium.

Results: Population weighted QALE estimates across LAs in GB were lowest in Scotland (females/males: 65.1y/64.9y) and Wales (65.0y/65.2y), while they were highest in England (67.5y/67.6y). The range across LAs for females was from 56.3y (80% CI: 45.6 to 67.1) in Mansfield to 77.7y (65.11 to 90.2) in Runnymede. QALE for males ranged from 57.5y (40.2 to 74.7) in Merthyr Tydfil to 77.2y (65.4 to 89.1) in Runnymede. Indicators of the inclusive economy accounted for more than half of the variation in QALE at the LA level (adjusted R-squared females/males: 50%/57%). Although more inclusivity was generally associated with higher levels of QALE at the LA level, this association was not consistent across all 13 inclusive economy indicators.

Conclusions: QALE can be estimated for LAs in GB, enabling further research into area-level health inequalities. The associations we identified between inclusive economy indicators and QALE highlight potential policy priorities for improving population health and reducing health inequalities.

For peer review only - http://bmjopen.bmj.com/site/about/guidelines.xhtml

Strengths and limitations of this study

- We used a small-area approach to model mortality rates and health state utility scores - the two components required to estimate quality-adjusted life expectancy (QALE).
- The small-area approach described is suitable for estimating other health and state expectancy measures, and is easily transferable to other national contexts for which similar data are available.
- As with most measures of health expectancy for small areas, QALE point estimates come with some degree of uncertainty, primarily due to their reliance on survey data.
- Differences in sampling strategies and non-response bias between local authorities in the Understanding Society survey might have contributed to differences in health state utility scores between areas.

For peer review only

Introduction

Period life expectancy (LE) is a summary measure of age-specific mortality rates, reflecting a particular point in time [1]. A limitation of LE is that it does not include any assessment of health. Health expectancy measures seek to address this limitation and capture both length of life and health status.

A variety of health expectancy measures are available for research and public health planning, including healthy life expectancy (HLE), disability-free life expectancy (DFLE) – and more recently quality-adjusted life expectancy (QALE). All three measures of health expectancy use life tables to quantify the length of life [2,3]. However, these three health expectancy measures differ substantially in how they measure the health status of individuals. HLE and DFLE measure health in binary terms, by capturing individual-level health at every age as a value of either 1 *or* 0. HLE and DFLE reflect a single dimension of health (either “healthy” vs. ”unhealthy” or “without disability” vs. ”with disability”). QALE captures health in far greater detail using individual-level health state utility scores (hereafter: utility scores).

Individual-level utility scores measure health at every age as a value *between* 0 (dead) and 1 (perfect health) [3]. Typically, scores are estimated from standardised questionnaires such as the EuroQol 5-dimension (EQ-5D) or the short form 12 (SF-12) tools - all of which capture a range of health dimensions [4,5]. Due to this greater detail, QALE captures both mental and physical health and is more sensitive to changes in health that would typically be masked by the binary definitions used for HLE or DFLE. This places QALE conceptually close to the gold standard measure, quality-adjusted life years (QALYs), which is the standard metric used in health economics to evaluate cost effectiveness [6]. While QALYs are typically referred to as an outcome measure in longitudinal study designs, QALE reflects the application of the underlying concept of utility scores to a synthetic life table cohort - analogously to the distinction enabled by other measures of health expectancy (e.g., disability-free life years (DFLY) vs. DFLE).

A growing number of studies have used QALE and QALYs to estimate the impact of shocks and interventions on population health. For example, the concepts have been

For peer review only - http://bmjopen.bmj.com/site/about/guidelines.xhtml

used as an outcome measure to understand the health burden of COVID-19 [7] and to shed light on the health impact of diseases [8,9] or adverse life course events [10]. The concepts are also used in simulation studies to evaluate health technologies and policies [11]. Despite the concept's increasing popularity, QALE estimates are often not available for small areas. While QALE has been estimated for the general English population [12], and broken down by deprivation quintile [13], no QALE estimates are available for other GB nations or GB local authorities (LAs). In this paper, we present a small-area approach for estimating QALE for LAs (lower tier/district level) in England, Scotland, and Wales.

Despite continued economic growth, GB has experienced a stalling of improvements in mortality and health, alongside widening area-level inequalities [14]. In response, creating a more inclusive economy has increasingly gained traction as a suitable framework to tackle health inequalities. In an exploratory analysis, we therefore examined potential factors accounting for inequalities in QALE at the LA level using 13 inclusive economy indicators, operationalised by the System Science in Public Health and Health Economics Research (SIPHER) consortium. SIPHER is a multi-disciplinary group of scientists and government partners at local, regional, and national levels [15]. Focusing on a range of different domains, the concept of the inclusive economy approaches the extent and nature of economic inclusion and participation. As economies are re-built following the COVID-19 pandemic, the concept of the inclusive economy might be more important for explaining population health outcomes and health inequalities than measures of economic growth or output [16].

Data & Methods

Estimating Life Tables

QALE requires information on two components: mortality and health. To derive the mortality component, we estimated period life tables based on mid-year population estimates and death counts. Mid-year population estimates for all three GB nations, as

For peer review only - http://bmjopen.bmj.com/site/about/guidelines.xhtml

well as death counts for England and Wales, were accessed via the Office for National Statistics' (ONS) web platform NOMIS. We accessed NOMIs via its underlying programming interface (API). Death counts for Scottish LAs in the respective period were obtained from the website of National Records of Scotland (NRS). For both the mortality and health components of QALE, we consistently applied the ONS' definition of LA boundaries as of April 2021. We pooled data for the years 2018, 2019 and 2020.

Despite pooling data for three consecutive years, estimating robust age-specific mortality rates for an area with a small population size is challenging due to small and fluctuating numbers [17,18]. The potential imprecision in age-specific mortality rates increases as the size of the area decreases and fewer deaths are recorded [19]. One approach to address this challenge is to apply specific small-area estimation techniques, such as the tool for projecting age-specific rates using linear splines (TOPALS) models.

TOPALS models have previously been used to estimate mortality rates for small areas [20–23]. Given the general shape of a reference age pattern, TOPALS models estimate age-specific mortality rates by using penalised iteratively reweighted least squares (PIRLS). Reference age patterns should allow for smooth patterns [17] and align to the respective application [23]. We used data from the Human Mortality Database (HMD) for the general populations of England and Wales, and Scotland in the period 2010 to 2019 as a reference mortality schedule [24]. We corrected for potential over- or under-estimation of mortality after age 90 [25] - a phenomenon which can occur due to the functional form of TOPALS models [23].

We followed standard methodology to derive life tables [26]. An example of sparse mortality data for LAs with small population sizes (e.g., males from the Shetland Islands) is presented in **Figure 1**. **Figure 1** shows raw death counts and raw death rates for 5-year age groups - as well as the modelled mortality rates for 1-year age groups.

[FIGURE 1 here]

For peer review only - http://bmjopen.bmj.com/site/about/guidelines.xhtml

Estimating Utility Scores

To derive the health component of QALE, we estimated age- and sex-specific utility scores using a special license area linkage of the Understanding Society survey. Expressed on a scale *between* 0 (dead) and 1 (perfect health), utility scores summarise an individual's health by combining information on physical and mental health dimensions in one number [3,27]. Understanding Society is a large-scale, randomly sampled, panel survey which is representative of the UK population [28]. A special license was required to identify the LA of each interviewed household. We used wave 10 ("*j*"), which covered the period 2018-2020, the same period for which we estimated life tables.

In Understanding Society, the SF-12 (Version 2) measure is used to capture the health of individuals. For each survey participant in Understanding Society, the SF-12 provides a summary score for the physical (PCS) and mental health (MCS) status. In wave 10 ("*j*"), SF-12 data were available for 16,481 females and 13,118 males.

Using cross-sectional survey weights, we calculated raw age-specific mean PCS scores and MCS scores, separately for males and females of each LA. However, as these raw age- and sex-specific mean PCS scores and MCS scores were generally noisy and not always available for all ages among some LAs with very low numbers of respondents, we applied a modelling approach.

Using two weighted linear regression models, we predicted age-specific mean SF-12 PCS scores and MCS scores for males and females in each LA from previously calculated raw age-and sex-specific mean PCS scores and MCS scores. Both models included covariates for: *sex* (female/male), *age* (categorical 10-year age groups allowing for non-linear age-trends), an *interaction effect between sex and age*, and a *fixed LA-effect* (for each LA). We chose this functional form as it balanced statistical fit and over-fitting. All males and females aged 19 or younger were combined in the age group *0-19*. While the number of respondents varied across LAs, sample sizes were always sufficient for a fixed LA-effect to be estimated.

For peer review only - http://bmjopen.bmj.com/site/about/guidelines.xhtml

We converted all modelled age-and sex-specific mean SF-12 PCS scores and MCS scores into age-and sex-specific mean utility scores. For this purpose, we used the six-variable predictive model outlined by Lawrence and Fleishman [29] to map SF-12 PCS scores and MCS scores to utility scores. Conversions of SF-12 PCS scores and MCS scores into utility scores, via mapping, are common in health economics research with previous studies showing that mapping approaches generally perform well [30]. **Figure 2** shows the age- and sex-specific utility scores we estimated, and highlights examples for some of the largest LAs in GB and where authors were able to sense-check results with SIPHER's local and national policy partners.

[FIGURE 2 here]

Quantifying Uncertainty of QALE Estimates

Our LA-level QALE estimates are subject to some degree of uncertainty. To quantify the magnitude of this uncertainty, we have estimated confidence intervals (CIs) for all point estimates.

Approached via the Sullivan method, the uncertainty of health expectancies arises from the sum of its two component parts: the health and the mortality components [31]. We estimated the uncertainty of QALE point estimates considering these two components and as described by Jagger et al. 2014 [31]. While the variation of the health component reflects the uncertainty of the utility scores due to the number of survey participants, the variation of the mortality component reflects the uncertainty of estimated death rates and resulting death counts in the population which we obtained via TOPALS models. Across all LAs, the number of survey participants was substantially lower when compared to the actual population size of the respective LA. As a result, the overall amount of uncertainty is clearly dominated by the amount of uncertainty arising from the health component.

For peer review only - http://bmjopen.bmj.com/site/about/guidelines.xhtml

We provide 80% CIs, rather than 95% CIs for our point estimates. 80% CIs lead to smaller ranges and reflect 1.282 times the standard deviation as opposed 1.960 times the standard deviation. Using 80% CIs represents a compromise between uncertainty and usefulness with respect to our LA-level QALE estimates. 80% CIs have previously been used for health expectancy estimates for national, regional, and super-regional levels [32].

Combining Life Tables and Utility Scores to Estimate QALE

We estimated QALE at birth in years for males and females for each LA using the Sullivan method [33]. The Sullivan method represents an adjustment of the life table parameter *nLx*, the number of person-years lived between age *x* and *x+1*, by the utility score of the age group *x*. Due to a very small number of respondents in the Understanding Society survey from the LAs City of London and Isles of Scilly, no meaningful QALE estimates could be derived for these LAs. We excluded these areas from all analyses. This reduced the number of LAs for which QALE was estimated to 361 out of 363 LAs in GB.

Indicators of the Inclusive Economy

To identify potential factors accounting for inequalities in QALE, we examined the association between indicators of the inclusive economy and QALE. For this purpose, we merged the obtained area-level QALE point estimates with the 13 inclusive economy indicators included in the SIPHER Inclusive Economy (local authority level) data set - an open access data set developed by the SIPHER consortium [34].

To date, there is no single definition of the inclusive economy, making the concept challenging to capture. In response, SIPHER has created an inclusive economy indicator set for all 363 LAs in GB to provide a meaningful collection of data that can be used to explore the extent and nature of economic inclusion. Details of the iterative and stakeholder process for selecting final indicators have previously been described in a

For peer review only - http://bmjopen.bmj.com/site/about/guidelines.xhtml
For peer review only

technical report [35]. From the Inclusive Economy (local authority level) data set, we used data for the year 2019, the midpoint of our three-year study period. A descriptive overview of all 13 indicators used in this study is presented in **Table 1**.

[TABLE 1 here]

We examined the association between indicators of the inclusive economy and QALE using multiple linear regression models. To ensure comparability across all indicators, we used a z-transformation to standardise regression coefficients.

All analyses were performed in R (Version 4.3.2). NOMIS API queries were carried out using the NOMISR package [36]. TOPALS models followed the approach and software provided by Schmertmann & Rau [23,37].

Ethical Approval

The University of Essex Ethics Committee has approved all data collection for the Understanding Society survey and the specific special license linkage. We obtained a special license version of Understanding Society (PN 228941). No additional ethical approval was necessary for other data sources or analyses.

Patient and Public Involvement

We did not engage with patients and the public to comment on study design, define outcomes, conduct analyses, interpret results, or write the manuscript. However, co-production within the consortium, involving SIPHER's policy and practice partners, has contributed to different elements of our work on identifying and discussing indicators of the inclusive economy and measures of population health. Results presented in this paper were presented and discussed at workshops with SIPHER's academic and policy

For peer review only - http://bmjopen.bmj.com/site/about/guidelines.xhtml

partners. We will continue to disseminate results to our partners at local, regional, and national levels.

Results

National Differences in QALE

QALE differed between GB nations when comparing population-weighted averages across LAs. QALE was lowest in Scotland at levels of 65.1 years (y) among females and 64.9y among males. Levels were slightly higher in Wales with 65.0y among females and 65.2y among males. QALE was highest in England at levels of 67.5y among females and 67.6y among males.

Differences in QALE Between Local Authorities in Great Britain

Visualising urban and rural areas with different population densities is challenging. Topographical maps can be dominated by large rural areas, overshadowing small but densely populated areas and clustering into higher-level geographies. We therefore used hexagon cartograms to visualise the area-level distribution of QALE for females and males separately (**Figure 3**) [38]. This approach visually accounts for the population size of areas, while retaining their contiguity and allowing for clustering into higher-level geographies. A detailed topographical map is presented in **Supplementary Figure 1**.

For females in Scotland, we found QALE to be lowest in North Ayrshire at a level of 60.0y (80% CI: 51.8 to 68.2) and highest in Na h-Eileanan Siar at a level of 74.1y (53.5 to 94.7). Among males in Scotland, QALE was lowest in Renfrewshire at a level of 59.6y (51.7 to 67.5), while it was highest in Na h-Eileanan Siar at a level of 72.7y (51.1 to 94.4). This resulted in a difference of 13.1y (among females) and 16.1y (among

For peer review only - http://bmjopen.bmj.com/site/about/guidelines.xhtml

males) in QALE point estimates across the Scottish LAs with the highest and the lowest level of QALE.

For females and males in Wales, we found QALE to be lowest in Merthyr Tydfil at levels of 56.9y (41.4 to 72.3) and 57.5y (40.2 to 74.7), while QALE was highest in Vale of Glamorgan at levels of 71.4y (64.6 to 78.3) and 71.4y (65.4 to 77.3), respectively. The corresponding difference in QALE point estimates between Welsh LAs with the highest and the lowest QALE was 14.5y among females and 13.9y among males.

Among females and males in England, QALE was lowest in Mansfield at levels of 56.3y (45.6 to 67.1) and 57.7y (48.2 to 67.2), while it was highest in Runnymede at levels of 77.7y (65.1 to 90.3) and 77.2y (65.4 to 89.1). Differences between the LAs with the highest and lowest QALE point estimates were the most striking in England at levels of 21.4y among females and 19.5y among males.

Sex Differences in QALE within Local Authorities

When looking at differences between females and males within the same LA, there was always a clear female advantage in LE. However, there was not always a clear female advantage in QALE. For example, females in Glasgow had a 5-year higher LE than males. The corresponding female QALE advantage was less than one year in Glasgow: 62.0y (57.4 to 66.7) among females and 61.2y (57.1 to 65.2) among males. For Sheffield, we found a female advantage in LE of approximately four years. However, there was a small disadvantage in QALE for females (63.4y (60.3 to 66.4)) when compared to males (63.6y (60.8 to 66.4)).

Associations between Inclusive Economy Indicators and QALE

We examined the associations between the inclusive economy indicators and QALE to gain insights into potential factors contributing to QALE inequalities at the LA-level in GB (**Figure 4**). Overall, we found that inclusive economy indicators explained more than half of the variation in QALE between LAs in GB (adjusted R-squared females/males:

For peer review only - http://bmjopen.bmj.com/site/about/guidelines.xhtml

For peer review only

50%/57%). We hypothesised that indicators reflecting more inclusivity would be associated with higher levels of QALE and that indicators reflecting lower levels of inclusivity would be associated with lower levels of QALE. As shown in **Figure 4**, our results were mixed as the direction of associations and levels of significance were not always consistent with our hypotheses.

For 6 out of the 13 inclusive economy indicators, we found the direction of the association between indicator and QALE to be in line with our hypotheses (more inclusive: higher QALE). For example, in relation to the observed mean level of public physical connectivity for the whole of GB (62.50%), an increase by 1 standard deviation (28.02%) was associated with an increase in QALE among females by 0.36y (95% CI: -0.02 to 0.74) and among males by 0.49y (95% CI: 0.14 to 0.85). Consistent with our hypotheses (less inclusive: lower QALE), we also found that an increase in digital exclusion by 1 standard deviation (20.22%), in relation to the observed GB mean level (33.86%), was associated with a decrease in QALE among females by 1.34y (95% CI: -1.91 to -0.78) and among males by 1.44y (95% CI: -1.97 to -0.91).

For the remaining 7 out of the 13 inclusive economy indicators, the direction of the association between indicator and QALE was not in line with what we hypothesised. For example, we found that an increase in job security was associated with a decrease in QALE among females (-0.23y; 95% CI: -0.53 to 0.06) and among males (-0.23y; 95% CI: -0.50 to 0.05)

One potential mechanism behind unexpected patterns could be residual confounding. For example, with respect to job security, non-permanent employment could be associated with well-paid occupations. High ratios of house prices to earnings (i.e., the metric used for the housing affordability indicator) might indeed capture less inclusivity, but could be indicative of affluent suburban areas.

For peer review only - http://bmjopen.bmj.com/site/about/guidelines.xhtml

Discussion

Principal Findings

This paper details an approach for estimating QALE for LAs in GB, enabling further research into area-level health inequalities. Our approach harnesses the power of existing data and is transferable to other national contexts for which required data on mortality and health are available. Results of our exploratory analysis highlight the importance of economic inclusion as a potential policy priority for improving population health and reducing population health inequalities in GB.

Strengths and Limitations

This is the first study to estimate QALE for all LAs in GB. Both the mortality and health components of QALE were derived via statistical modelling techniques, rather than being representative of raw input data only. We therefore consider our LA-level QALE estimates to be reliable, and our approach to be transferable to other national contexts for which respective small-area data are available.

As with life expectancy, and most measures of health expectancy such as HLE and DFLE, the estimation of QALE is based on a synthetic cohort approach. This means that our estimates provide timely cross-sectional snapshots - and should not be considered as precise forecasts of quality-adjusted life years that individuals are likely to experience. In the absence of shocks to population health, such as famines, epidemics, and wars, measures of life (and health) expectancy tend to underestimate the years of life (and years in good health) as societal and medical progress are not accounted for [39]. On another note, recent period effects such as the consequences of austerity measures [40], the COVID-19 pandemic [41], and the subsequent cost-of-living crisis [42] highlight that deteriorations in population health can occur throughout the life course of individuals.

For peer review only - http://bmjopen.bmj.com/site/about/guidelines.xhtml

The sensitivity of life and health expectancy to cross-sectional patterns of mortality and health means that our QALE estimates might have been impacted by the COVID-19 pandemic. The pandemic cannot have had an impact on the health component of our QALE estimates as the collection of survey data occurred throughout 2018 and 2019. However, the pandemic might have had an impact on the mortality component, which covers the years 2018 to 2020. As a sensitivity analysis, we therefore re-ran our analysis not considering mortality observed throughout the year 2020. A comparison of resulting differences in QALE is presented in **Supplementary Figure 2**. As expected, we found that excluding mortality of 2020 resulted in an (unweighted) increase of QALE of about 0.17 years among females and 0.28 years among males across all studied LAs.

We obtained QALE estimates based on the Sullivan method. As a prevalence-based approach, the Sullivan method stipulates one universal mortality pattern, via the *nLx* parameter, for all individuals represented by a life table. This is a major methodological drawback, as it is likely that mortality will differ across individuals of different health status [43].

As with all measures of health expectancy, especially when estimated for small areas, our QALE estimates are subject to a substantial level of uncertainty. This uncertainty is typically much larger than the uncertainty surrounding corresponding LE estimates [44]. As a result, care is required when comparing health expectancy point estimates in longitudinal analyses, in particular across areas with small population sizes. The uncertainty of our QALE estimates arises from the relatively small number of survey respondents contributing to the estimation of the health component. We have decided to not further increase the survey sample size synthetically when estimating the health component of QALE. For example, the survey sample size could have been synthetically increased to reflect the entire adult population in GB using a full-scale synthetic population data set which we have previously developed, and which is based on the Understanding Society survey [45]. Instead, we have relied on the original sample size of the survey, reflecting its special license area-level linkage. While the Understanding Society survey is representative of the UK population [28], sampling

For peer review only - http://bmjopen.bmj.com/site/about/guidelines.xhtml

strategies and non-response will likely have varied across LAs, deprivation groups, and by health status. Weighting and oversampling might have not fully corrected for these limitations.

To estimate life tables, we used official LA-level mid-year population estimates which are rolled forwards from the preceding census, using a cohort component model. The estimates based on the 2011 census have recently been revised to align with the 2021 census in England and Wales, but an equivalent adjustment has yet to be made in Scotland. Irrespective of adjustments, unrecorded international migration, and migration between LAs could have had an impact on the quality of mid-year population estimates, affecting the estimation of age- and sex-specific mortality rates. As the civil registration system in GB does not allow us to follow individuals' place of residence between censuses, this numerator-denominator issue, alongside differences in data availability for the three GB nations, is likely to remain an unresolved challenge for periods between censuses [46].

We accessed data on mid-year population estimates and death counts for 5-year age groups via NOMIS. These routinely provided data included rounded mid-year population estimates (to 100s), while death counts below 5 were disclosed for LAs in England and Wales. NOMIS data can differ in comparison to bespoke data access requests available via the main ONS website. While bespoke data requests may contain more detailed information, they are not routinely updated - unless re-requested. To maximise reproducibility, transferability, and updateability of the underlying pipeline, we queried data in code-based format via NOMIS and its API. This reflects a sustainable and continuously updated source of data. Due to the use of TOPALS models for modelling mortality rates against the general population standard, the overall impact of differences across different data sources is likely to be negligible

For peer review only - http://bmjopen.bmj.com/site/about/guidelines.xhtml

Implications of Findings and Relevance to Other Studies

When aggregated and weighted by the population of each LA, our QALE estimates for England are slightly lower than previously reported QALE estimates for the English population in 2017-2018 (females: 67.5y vs. 68.2y; males: 67.6y vs. 68.2y) [12]. Small differences in values were expected and could be due to a multitude of aspects: differences in the sampling biases in the survey data used, the measure used to capture health (EQ-5D vs. SF-12v2), or the exact years reflected. At the same time, this difference could reflect the observed stall in population health improvements across GB (e.g., [40]).

Findings of our analysis indicate large inequalities in QALE across LAs in GB, further evidencing substantial area-level inequalities in population health. Our findings for QALE show several similarities when compared with patterns reported for HLE across LAs in GB reported for HLE across LAs in GB [47,48]. For example, in line with results for HLE, the difference in QALE between areas with the highest and the lowest levels was much larger than the difference with respect to LE. This is further evidence that capturing mortality in isolation is likely to underestimate the differences in the burden of ill health experienced across GB.

Generally, we found areas with low levels of QALE to also be among the lowest with respect to HLE. For example, we estimated QALE to be the lowest among males in Merthyr Tydfil at levels of 57.5y while HLE in the respective period was also low at a level of 58.9y [47]. Nevertheless, differences in how health is captured means that absolute levels and rankings will likely vary across different measures of health expectancy. At the same time, researchers and policymakers would benefit from developing a stronger comparative approach which captures the performance of areas with respect to different population health metrics [49].

When looking within the same LA, females always had higher levels of LE than males. In line with patterns for other health expectancy measures [50] and previously reported results for QALE across population subgroups in England [51], we found the female advantage in QALE to be very small, or negligible. This represents a health-survival

For peer review only - http://bmjopen.bmj.com/site/about/guidelines.xhtml

paradox: Females live longer but do not necessarily have better health in comparison to males. It is still not fully understood whether this health-survival paradox is the reflection of a true female health disadvantage (despite having a survival advantage), or the result of gender differences in self-reporting health [52]. It is possible that the health component of QALE could be affected by sex-specific survey response behaviour. Sex differences in QALE could stem from sex differences in the SF-12 health instrument which we used to derive utility scores. The SF-12 health instrument captures mental health explicitly. Averaged across LAs, we found the female disadvantage in the SF-12 mental health score (females: 46.8 vs. males: 49.2, $p<0.01$) to be larger than the female disadvantage in the SF-12 physical health score (females: 48.7 vs. males: 49.7, $p<0.01$).

Historically, improvements in population health were often attributed to economic growth [53]. However, this assumption is increasingly contested for high-income countries, including GB, as the association between economic growth and improving health does not seem to hold past certain levels of development [14,40]. In response, there have been growing calls to shift policy ambitions away from economic growth, towards inclusive economies, with a view to achieving more sustainable and equitable health improvements [16]. We found that inclusive economy indicators explained more than half of the variation in QALE between LAs in GB. This highlights the importance economic inclusion may have for improving population health and reducing area-level health inequalities. At the same time, associations found between inclusive economy indicators and QALE were not always consistent with our hypotheses, highlighting the concept's complexity. One alternative approach would be to collapse information on all indicators into one single, averaging summary indicator, as it is often done with deprivation indices. However, one averaged summary indicator will likely fail to capture the diversity of inclusive economy indicators and the variation across different urban and rural areas. Here, more work is needed to refine and test how the inclusive economy can be captured.

For peer review only - http://bmjopen.bmj.com/site/about/guidelines.xhtml

For peer review only

Conclusion

QALE is a comprehensive health expectancy measure and can be estimated for small areas in GB. QALE is conceptually close to the concept of QALYs, making it a comprehensive outcome measure for approaching area-level health inequalities, and evaluating past and future policies such as those seeking to establish more inclusive economies.

For peer review only

For peer review only - http://bmjopen.bmj.com/site/about/guidelines.xhtml

Tables

Table 1: Descriptive overview of quality-adjusted life expectancy (QALE) at birth, life expectancy (LE) at birth, and inclusive economy indicators for all local authorities (LAs) in England, Scotland, and Wales studied (n=361 LAs, meaning it does not consider the LAs City of London and Isles of Scilly). Note: Values in this table provided for the mean are representative of raw, unweighted averages across all studied LAs. In addition, please note the direction of indicators. For example, a higher employment rate is considered favourable, while a higher proportion of child poverty is not favourable. As some of the indicators reflect ratios, we did not change the direction of indicators. We utilised the direction provided in the underlying inclusive economy indicator set.

Indicator	Mean	SD	Min	Max	Explanation
LE Male	79.68	1.92	73.37	84.19	Life expectancy
QALE Male	67.88	3.83	57.46	77.23	Quality-adjusted life expectancy
LE Female	83.42	1.62	78.42	86.76	Life expectancy
QALE Female	67.70	3.84	56.34	77.72	Quality-adjusted life expectancy
Participation in paid employment	76.67	5.09	61.80	89.10	Percentage of working-age population (age 16-64) who are employed.
Skills and qualifications	73.89	9.54	37.65	93.92	Percentage of adults aged 16-49 with a Level 2 or higher National Vocational Qualification (NVQ).
Involuntary exclusion from the labour market	4.97	2.15	1.05	11.74	Percentage of working-age population (age 16-64) who are economically inactive due to ill health or disability.
Digital exclusion	33.86	20.22	0.00	87.23	Percentage of individuals who are classified as e-withdrawn, passive, and uncommitted internet users,

For peer review only - http://bmjopen.bmj.com/site/about/guidelines.xhtml

Indicator	Mean	SD	Min	Max	Explanation
					or settled offline communities, based on the Internet User Classification (IUC).
Wealth inequality	2.68	1.06	1.31	9.30	Ratio of max to min house sale prices within each local authority
Physical connectivity	62.50	28.02	0.00	100.00	Proportion of LSOAs/DZs within the local authority area that are among the 50% most accessible LSOAs/DZs for each devolved nation (56%/44% split in England).
Earnings inequality	3.13	0.39	2.29	5.51	Ratio of weekly earnings (residents in full-time work) between 80th and 20th percentiles.
Housing affordability	9.92	3.62	3.95	34.46	Ratio of median house prices to median gross annual earnings (for residents) within each local authority.
Poverty	27.54	7.07	12.50	55.41	Percentage of children living in low-income households (after adjustment for housing costs).
Cost of living	10.83	2.59	5.56	21.63	Percentage of individuals reporting that they are worried about getting the food they need.
Decent Pay	21.77	6.06	7.50	40.40	Percentage of employee jobs that are not paid at or above the living wage.
Inclusion in	35.18	6.19	21.60	64.47	Percentage of individuals

For peer review only - http://bmjopen.bmj.com/site/about/guidelines.xhtml

Indicator	Mean	SD	Min	Max	Explanation
decision-making					participating in local elections out of all eligible voters.
Job security	95.31	1.69	86.13	98.33	Percentage of individuals in permanent employment out of all employed individuals aged 16-64.

For peer review only

Contributorship statement: AH provided a first draft of the formal analysis and paper. AH, NL, HR and CH curated the underlying data. AH, NL, HR and CH contributed to the formal data analysis and validation of results. AH, NL, HR, CA, AB, DB, AC, CE, CH, SVK, GMC, RS, AT and PM contributed to conceptualisation, study design, interpretation of findings, and the discussion of implications. All authors contributed to the first draft of the paper, the revision of the paper, and have approved the final version. PM is the overall guarantor of this work.

Competing interests: no competing interests

Funding: This work was supported by the UK Prevention Research Partnership (MR/S037578/2), which is funded by the British Heart Foundation, Cancer Research UK, Chief Scientist Office of the Scottish Government Health and Social Care Directorates, Engineering and Physical Sciences Research Council, Economic and Social Research Council, Health and Social Care Research and Development Division (Welsh Government), Medical Research Council, National Institute for Health Research, Natural Environment Research Council, Public Health Agency (Northern Ireland), The Health Foundation and Wellcome. In addition, this project received funding from the Medical Research Council (MC_UU_00022/5) as well as the Chief Scientist Office (SPHSU17 and SPHSU20). SVK acknowledges funding from the European Research Council (949582), and the Medical Research Council (MC_UU_00022/2). The funders had no role in the design of the study or in the collection, analysis, and interpretation of data and results.

Data sharing statement: We created a reproducibility pack for this paper: *[DOI provided after the peer review process]* The reproducibility pack contains detailed results as well as all code and data which we are able to share as open access material. We have published indicators of the inclusive economy as a separate data set: https://doi.org/10.17605/OSF.IO/VNSUR

For peer review only - http://bmjopen.bmj.com/site/about/guidelines.xhtml

References

1 Modig K, Rau R, Ahlbom A. Life expectancy: What does it measure? *BMJ open*. 2020;10:e035932.

2 Gold MR, Stevenson D, Fryback DG. HALYS and QALYS and DALYS, oh my: Similarities and differences in summary measures of population health. *Annu Rev Public Health*. 2002;23:115–34.

3 Prieto L, Sacristán JA. Problems and solutions in calculating quality-adjusted life years (QALYs). *Health Qual Life Outcomes*. 2003;1:1–8.

4 Sullivan PW, Slejko JF, Sculpher MJ, *et al.* Catalogue of EQ-5D scores for the United Kingdom. *Med Decis Making*. 2011;31:800–4.

5 Brazier JE, Roberts J. The estimation of a preference-based measure of health from the SF-12. *Medical Care*. 2004;851–9.

6 Whitehead SJ, Ali S. Health outcomes in economic evaluation: The QALY and utilities. *British medical bulletin*. 2010;96:5–21.

7 Wouterse B, Ram F, Baal P van. Quality-adjusted life-years lost due to COVID-19 mortality: Methods and application for the Netherlands. *Value Health*. 2022;25:731–5.

8 Brønnum-Hansen H, Juel K, Davidsen M, *et al.* Impact of selected risk factors on quality-adjusted life expectancy in Denmark. *Scand J Public Health*. 2007;35:510–5.

9 Jia H, Zack MM, Thompson WW. The effects of diabetes, hypertension, asthma, heart disease, and stroke on quality-adjusted life expectancy. *Value Health*. 2013;16:140–7.

10 Jia H, Lubetkin EI. Impact of adverse childhood experiences on quality-adjusted life expectancy in the U.S. population. *Child Abuse Negl*. 2020;102:104418.

11 The National Institute for Health and Care Excellence. NICE health technology evaluations: The manual. Process and methods [PMG36]. 2022.

For peer review only - http://bmjopen.bmj.com/site/about/guidelines.xhtml

For peer review only

https://www.nice.org.uk/guidance/pmg36/resources/nice-health-technology-evaluations-the-manual-pdf-72286779244741

12 McNamara S, Schneider PP, Love-Koh J, *et al.* Quality-adjusted life expectancy norms for the English population. *Value Health.* 2023;26:163-69.

13 Love-Koh J, Asaria M, Cookson R, *et al.* The social distribution of health: Estimating quality-adjusted life expectancy in England. *Value Health*. 2015;18:655-62.

14 Walsh D, Dundas R, McCartney G, *et al.* Bearing the burden of austerity: How do changing mortality rates in the UK compare between men and women? *J Epidemiol Community Health*. 2022;76:1027–33.

15 Meier P, Purshouse R, Bain M, *et al.* The SIPHER consortium: Introducing the new UK hub for systems science in public health and health economic research. *Wellcome open research*. 2019;4:174.

16 Shipton D, Sarica S, Craig N, *et al.* Knowing the goal: An inclusive economy that can address the public health challenges of our time. *J Epidemiol Community Health*. 2021;75:1129–32.

17 Raalte AA van. What have we learned about mortality patterns over the past 25 years? *Population Studies*. 2021;75:105–32.

18 Wilson T, Grossman I, Alexander M, *et al.* Methods for small area population forecasts: State-of-the-art and research needs. *Popul Res Policy Rev*. 2022;41:865–98.

19 Eayres D, Williams ES. Evaluation of methodologies for small area life expectancy estimation. *J Epidemiol Community Health*. 2004;58:243–9.

20 Beer J de. A new relational method for smoothing and projecting age-specific fertility rates: TOPALS. *Demographic Research*. 2011;24:409–54.

21 Beer J de. Smoothing and projecting age-specific probabilities of death by TOPALS. *Demographic Research*. 2012;27:543–92.

For peer review only - http://bmjopen.bmj.com/site/about/guidelines.xhtml

22 Gonzaga MR, Schmertmann CP. Estimating age- and sex-specific mortality rates for small areas with TOPALS regression: An application to Brazil in 2010. *Revista Brasileira de Estudos de População*. 2016;33:629–52.

23 Rau R, Schmertmann CP. District-level life expectancy in Germany. *Dtsch Arztebl Int*. 2020;117:493–9.

24 Barbieri M, Wilmoth JR, Shkolnikov VM, *et al.* Data resource profile: The Human Mortality Database (HMD). *Int J Epidemiol*. 2015;44:1549–56.

25 Kannisto V, Lauritsen J, Thatcher AR, *et al.* Reductions in mortality at advanced ages: Several decades of evidence from 27 countries. *Population and development review*. 1994;793–810.

26 Chiang CL. *Life table techniques and their applications*. Krieger Publishing Company 1984.

27 Kim YE, Jung YS, Ock M, *et al.* A review of the types and characteristics of healthy life expectancy and methodological issues. *J Prev Med Public Health*. 2022;55:1–9.

28 Buck N, McFall S. Understanding Society: Design overview. *Longitudinal and Life Course Studies*. 2011;3:5–17.

29 Lawrence WF, Fleishman JA. Predicting EuroQoL EQ-5D preference scores from the SF-12 health survey in a nationally representative sample. *Med Decis Making*. 2004;24:160–9.

30 Chuang L-H, Kind P. Converting the SF-12 into the EQ-5D: An empirical comparison of methodologies. *Pharmacoeconomics*. 2009;27:491–505.

31 Jagger C, Van Oyen H, Robine J-M. Health expectancy calculation by the Sullivan method: A practical guide. Nihon University Population Research Institute (NUPRI) Research Paper Series 68 (2014).

For peer review only - http://bmjopen.bmj.com/site/about/guidelines.xhtml

32 Permanyer I, Villavicencio F, Trias-Llimós S. Healthy lifespan inequality: Morbidity compression from a global perspective. *Eur J Epidemiol.* 2023;38: 511-521.

33 Sullivan DF. A single index of mortality and morbidity. *HSMHA Health Reports*. 1971;86:347.

34 [dataset] Rice H, Lomax N, Hoehn A, *et al.* Data from: SIPHER inclusive economy (local authority level) data set. Open Science Framework, December 14, 2023. https://doi.org/10.17605/OSF.IO/VNSUR

35 SIPHER. *The SIPHER inclusive economy indicator set: Technical paper.* SIPHER 2022. https://sipher.ac.uk/wp-content/uploads/2022/10/SIPHER-Inclusive-Economy-Indicator-set.pdf

36 Odell E. Nomisr: Access UK labour market data. *J Open Source Softw*. 2018;3:859.

37 Carl Schmertmann: TOPALS fitting with age-grouped data. Https://github.com/schmert/TOPALS. Accessed on: 2024-02-06.

38 House of Commons Library: Geographical templates for non-contiguous cartograms of the UK. https://github.com/houseofcommonslibrary/uk-hex-cartograms-noncontiguous. Accessed on: 2024-02-06.

39 Goldstein JR, Wachter KW. Relationships between period and cohort life expectancy: Gaps and lags. *Population Studies*. 2006;60:257–69.

40 Walsh D, Wyper GMA, McCartney G. Trends in healthy life expectancy in the age of austerity. *J Epidemiol Community Health*. 2022;76:743–5.

41 Aburto JM, Kashyap R, Schöley J, *et al.* Estimating the burden of the COVID-19 pandemic on mortality, life expectancy and lifespan inequality in England and Wales: A population-level analysis. *J Epidemiol Community Health*. 2021;75:735–40.

For peer review only - http://bmjopen.bmj.com/site/about/guidelines.xhtml

42 Höhn A, Stokes J, Pollack R, *et al.* Systems science methods in public health: What can they contribute to our understanding of and response to the cost-of-living crisis? *Journal of Epidemiology and Community Health*. 2023;77:610–6.

43 Shen T, Riffe T, Payne CF, *et al.* Decomposition of differentials in health expectancies from multistate life tables: A research note. *Demography*. 2023;60:1675–88.

44 Villavicencio F, Bergeron-Boucher M-P, Vaupel JW. Reply to Permanyer et al.: The uncertainty surrounding healthy life expectancy indicators. *Proc Natl Acad Sci.* 2021;118:e2115544118.

45 Wu G, Heppenstall A, Meier P, *et al.* A synthetic population dataset for estimating small area health and socio-economic outcomes in Great Britain. *Scientific Data*. 2022;9:19.

46 Leon DA, Shkolnikov VM. Widening life expectancy inequalities across small areas of England. *Lancet Public Health*. 2021;6:e783–4.

47 Office for National Statistics: Health state life expectancies, UK: 2018 to 2020. Https://www.ons.gov.uk/peoplepopulationandcommunity/healthandsocialcare/healthandlifeexpectancies/bulletins/healthstatelifeexpectanciesuk/2018to2020. Accessed on: 2024-02-06.

48 National Records of Scotland: Healthy life expectancy 2018-2020. Statistical report detailing healthy life expectancy (HLE) estimates for areas within Scotland including council areas and NHS health boards. 2022. https://www.nrscotland.gov.uk/files/statistics/healthy-life-expectancy/18-20/healthy-life-expectancy-18-20-report.pdf. Accessed on: 2024-02-06.

49 Collins B. Using a survey to estimate health expectancy and quality-adjusted life expectancy to assess inequalities in health and quality of life. *Value Health*. 2013;16:599–603.

For peer review only - http://bmjopen.bmj.com/site/about/guidelines.xhtml

50 Jagger C. EU and UK targets for healthy life expectancy–are they achievable? *Vienna Yearbook of Population Research*. 2021;19:15–22.

51 Collins B. Results from a well-being survey in the North West of England: Inequalities in EQ-5D–derived quality-adjusted life expectancy are mainly driven by pain and mental health. *Value Health*. 2017;20:174–7.

52 Oksuzyan A, Dańko MJ, Caputo J, *et al.* Is the story about sensitive women and stoical men true? Gender differences in health after adjustment for reporting behavior. *Social Science & Medicine*. 2019;228:41–50.

53 Omran AR. The epidemiologic transition: A theory of the epidemiology of population change. 1971. *Milbank Q*. 2005;83:731–57.

For peer review only

For peer review only - http://bmjopen.bmj.com/site/about/guidelines.xhtml

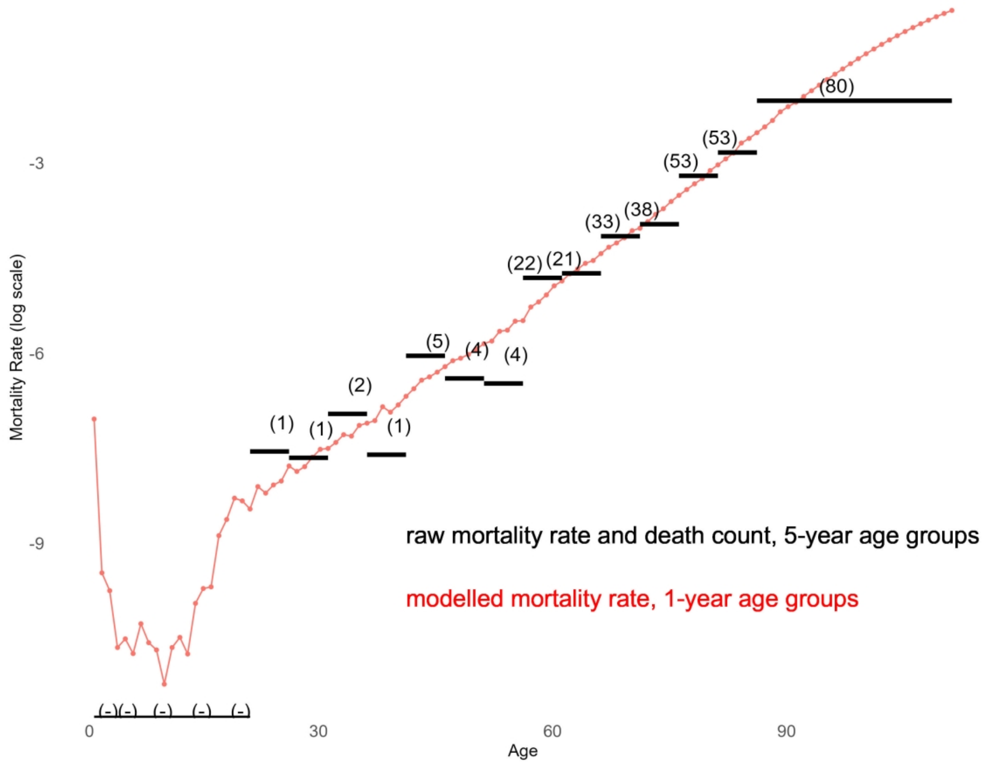

Figure 1: Number of observed deaths, the resulting raw death rate (log scale), and estimated mortality rates for males (log scale) of the Scottish local authority Shetland Islands, in 2018-2020.

1041x833mm (72 x 72 DPI)

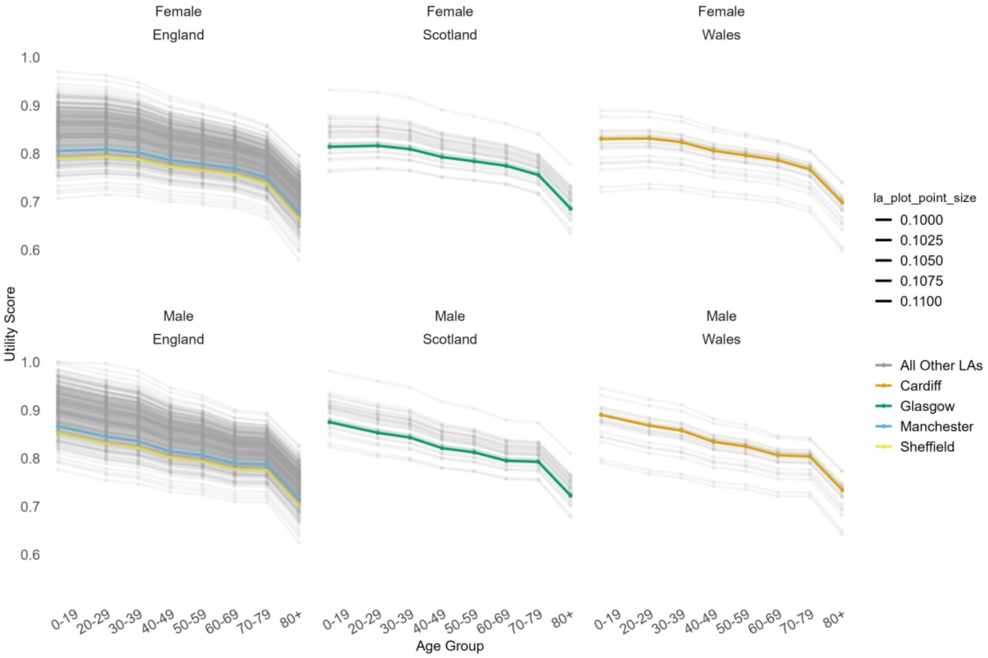

Figure 2: Utility scores obtained from SF12v2 PCS and MCS mean values for females and males in all local authorities in England, Scotland, and Wales with selected local authorities highlighted.

1249x833mm (72 x 72 DPI)

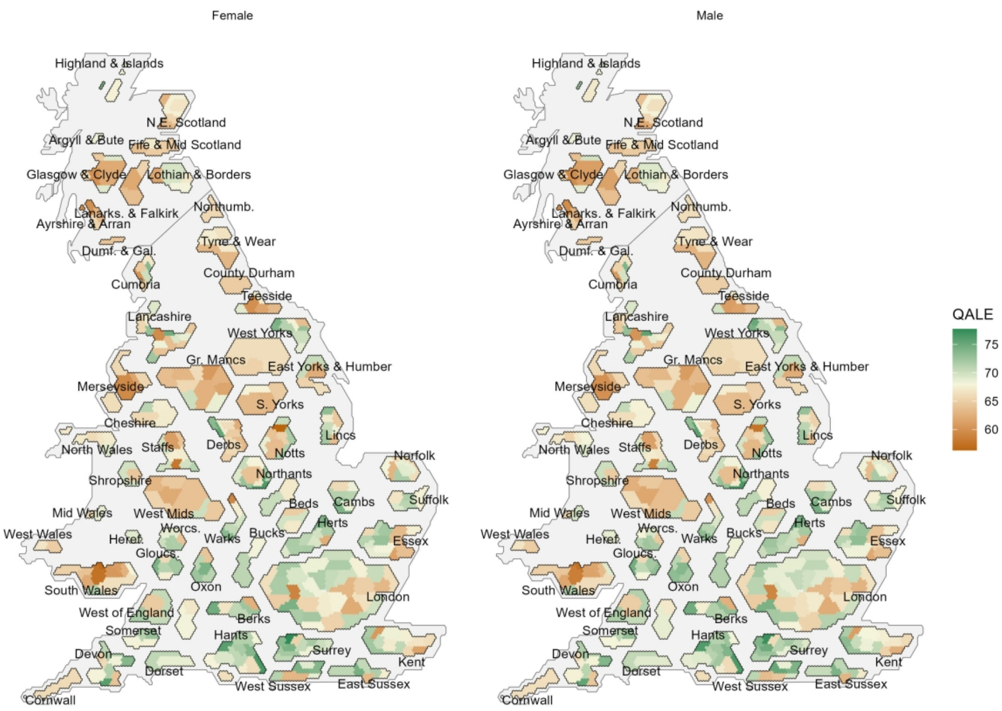

Figure 3: Quality-adjusted life expectancy (QALE) at birth in years in 2018-2020 for females and males in local authorities in England, Scotland, and Wales. Note: the mid-point of the colour scale is referring to the unweighted mean in QALE across all GB LAs.

1041x833mm (72 x 72 DPI)

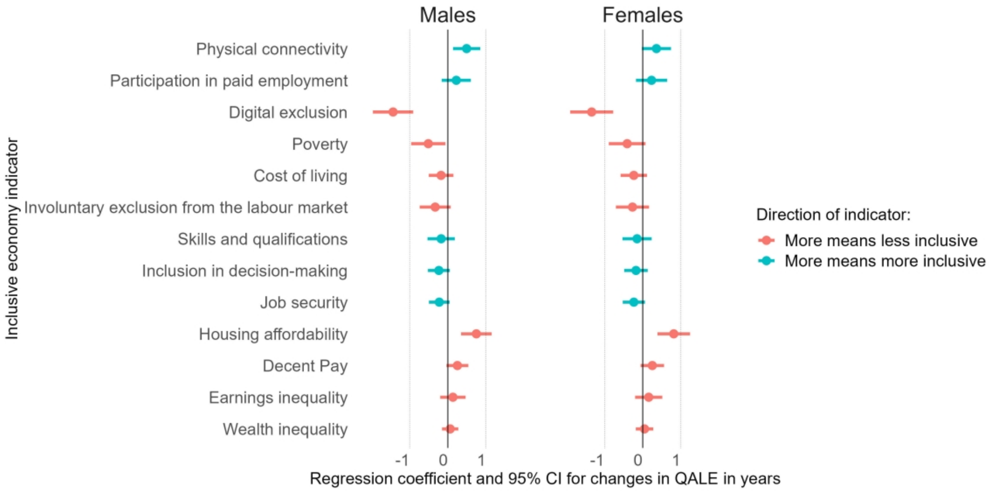

Figure 4: Multiple linear regression model for the association between inclusive economy indicators and quality-adjusted life expectancy (QALE) at birth in years and respective 95% confidence intervals (95% CIs). Note: regression coefficients were z-transformed. This means that regression coefficients show the expected change in QALE in years for a one standard deviation increase among the indicators, with respect to the GB mean level.

1249x624mm (72 x 72 DPI)

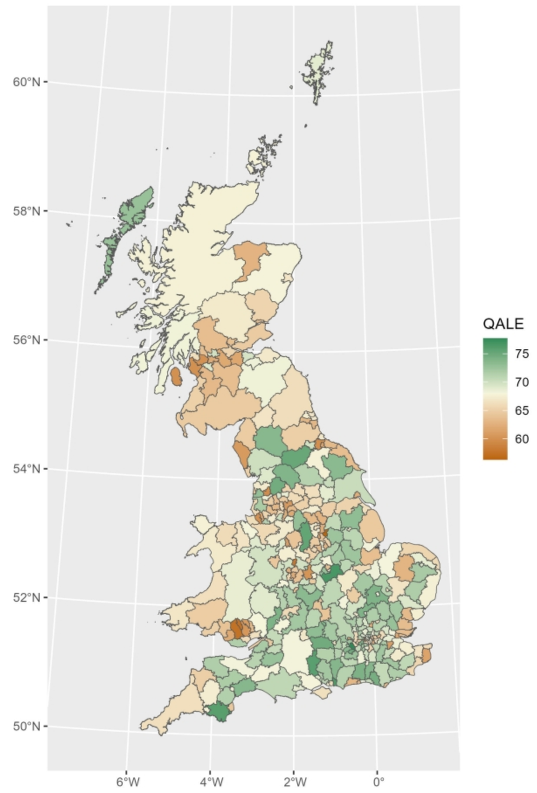

1041x833mm (72 x 72 DPI)

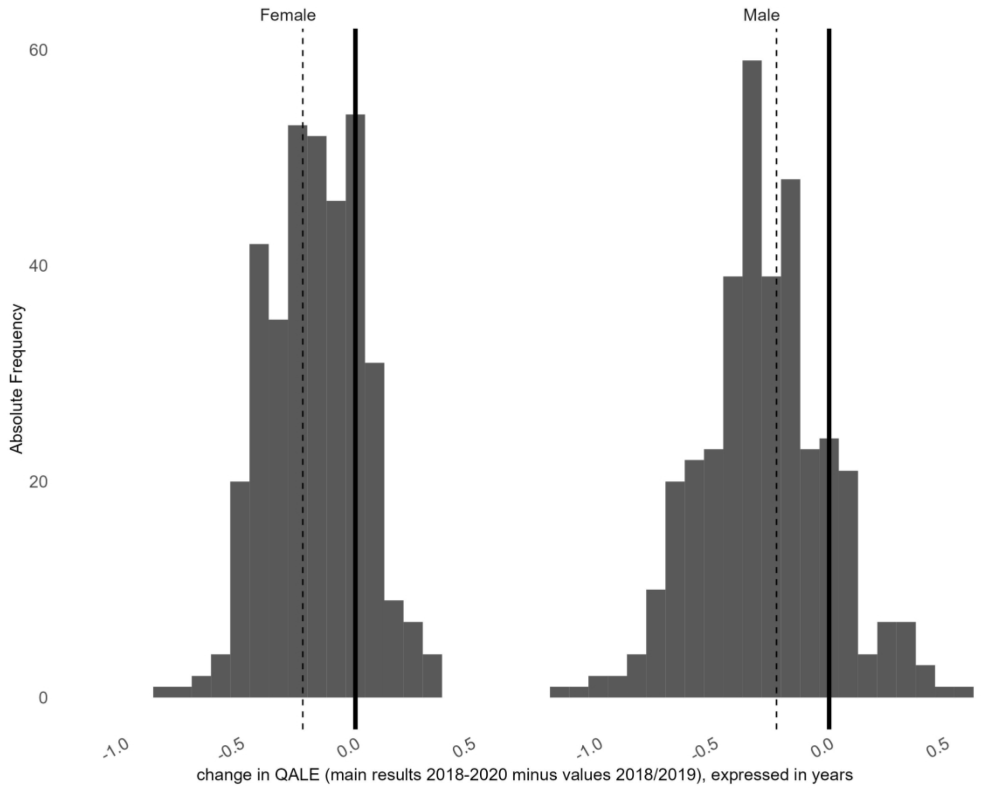

1041x833mm (72 x 72 DPI)

STROBE Statement—checklist of items that should be included in reports of observational studies

	Item No	Recommendation	Page No
**Title and abstract**	1	(*a*) Indicate the study's design with a commonly used term in the title or the abstract	1, 3
		(*b*) Provide in the abstract an informative and balanced summary of what was done and what was found	3
**Introduction**			
Background/rationale	2	Explain the scientific background and rationale for the investigation being reported	5-6
Objectives	3	State specific objectives, including any prespecified hypotheses	5-6
**Methods**			
Study design	4	Present key elements of study design early in the paper	6
Setting	5	Describe the setting, locations, and relevant dates, including periods of recruitment, exposure, follow-up, and data collection	6-10
Participants	6	(*a*) *Cohort study*—Give the eligibility criteria, and the sources and methods of selection of participants. Describe methods of follow-up *Case-control study*—Give the eligibility criteria, and the sources and methods of case ascertainment and control selection. Give the rationale for the choice of cases and controls *Cross-sectional study*—Give the eligibility criteria, and the sources and methods of selection of participants	6-10
		(*b*) *Cohort study*—For matched studies, give matching criteria and number of exposed and unexposed *Case-control study*—For matched studies, give matching criteria and the number of controls per case	NA
Variables	7	Clearly define all outcomes, exposures, predictors, potential confounders, and effect modifiers. Give diagnostic criteria, if applicable	6-11
Data sources/ measurement	8*	For each variable of interest, give sources of data and details of methods of assessment (measurement). Describe comparability of assessment methods if there is more than one group	*6-11*
Bias	9	Describe any efforts to address potential sources of bias	6-11
Study size	10	Explain how the study size was arrived at	6-11
Quantitative variables	11	Explain how quantitative variables were handled in the analyses. If applicable, describe which groupings were chosen and why	6-11
Statistical methods	12	(*a*) Describe all statistical methods, including those used to control for confounding	6-11
		(*b*) Describe any methods used to examine subgroups and interactions	6-11
		(*c*) Explain how missing data were addressed	6-11
		(*d*) *Cohort study*—If applicable, explain how loss to follow-up was addressed *Case-control study*—If applicable, explain how matching of cases and controls was addressed *Cross-sectional study*—If applicable, describe analytical methods taking account of sampling strategy	6-11
		(*e*) Describe any sensitivity analyses	NA

Continued on next page

For peer review only - http://bmjopen.bmj.com/site/about/guidelines.xhtml

**Results**			
Participants	13*	(a) Report numbers of individuals at each stage of study—eg numbers potentially eligible, examined for eligibility, confirmed eligible, included in the study, completing follow-up, and analysed	6-11
		(b) Give reasons for non-participation at each stage	NA
		(c) Consider use of a flow diagram	NA
Descriptive data	14*	(a) Give characteristics of study participants (eg demographic, clinical, social) and information on exposures and potential confounders	NA
		(b) Indicate number of participants with missing data for each variable of interest	NA
		(c) *Cohort study*—Summarise follow-up time (eg, average and total amount)	NA
Outcome data	15*	*Cohort study*—Report numbers of outcome events or summary measures over time	NA
		Case-control study—Report numbers in each exposure category, or summary measures of exposure	NA
		Cross-sectional study—Report numbers of outcome events or summary measures	12-14
Main results	16	(*a*) Give unadjusted estimates and, if applicable, confounder-adjusted estimates and their precision (eg, 95% confidence interval). Make clear which confounders were adjusted for and why they were included	12-14
		(*b*) Report category boundaries when continuous variables were categorized	12-16
		(*c*) If relevant, consider translating estimates of relative risk into absolute risk for a meaningful time period	NA
Other analyses	17	Report other analyses done—eg analyses of subgroups and interactions, and sensitivity analyses	16-19
**Discussion**			
Key results	18	Summarise key results with reference to study objectives	15
Limitations	19	Discuss limitations of the study, taking into account sources of potential bias or imprecision. Discuss both direction and magnitude of any potential bias	15-18
Interpretation	20	Give a cautious overall interpretation of results considering objectives, limitations, multiplicity of analyses, results from similar studies, and other relevant evidence	17-19
Generalisability	21	Discuss the generalisability (external validity) of the study results	15-19
**Other information**			
Funding	22	Give the source of funding and the role of the funders for the present study and, if applicable, for the original study on which the present article is based	24

*Give information separately for cases and controls in case-control studies and, if applicable, for exposed and unexposed groups in cohort and cross-sectional studies.

Note: An Explanation and Elaboration article discusses each checklist item and gives methodological background and published examples of transparent reporting. The STROBE checklist is best used in conjunction with this article (freely available on the Web sites of PLoS Medicine at http://www.plosmedicine.org/, Annals of Internal Medicine at http://www.annals.org/, and Epidemiology at http://www.epidem.com/). Information on the STROBE Initiative is available at www.strobe-statement.org.

For peer review only - http://bmjopen.bmj.com/site/about/guidelines.xhtml
